# Neolactotetraosylceramide enables urinary detection of bladder cancer

**DOI:** 10.1016/j.xcrm.2025.102246

**Published:** 2025-07-23

**Authors:** Inês B. Moreira, Charlotte Rossdam, Jonas Kaynert, Julia Beimdiek, Manuel M. Vicente, Jessica Schmitz, Anika Großhennig, Astrid Oberbeck, Michèle J. Hoffmann, Michele E. Rosero Moreno, Daniel Steinbach, Maria L. Barcena, Yannick Lippka, Jan H. Bräsen, Hossein Tezval, Falk F.R. Buettner

**Affiliations:** 1 Institute of Clinical Biochemistry, Hannover Medical School, 30625 Hannover, Germany; 2 Proteomics, Institute of Theoretical Medicine, Faculty of Medicine, University of Augsburg, 86159 Augsburg, Germany; 3 Institute for Pathology, Nephropathology Unit, Hannover Medical School, 30625 Hannover, Germany; 4 Institute of Biostatistics, Hannover Medical School, Carl-Neuberg-Straße 1, 30625 Hannover, Germany; 5 Department of Urology, Medical Faculty and University Hospital Düsseldorf, Heinrich Heine University Düsseldorf, 40225 Düsseldorf, Germany; 6 Department of Urology, Jena University Hospital, Friedrich-Schiller University, 07747 Jena, Germany; 7 Department of Urology, Eberhard Karls University of Tübingen, 72076 Tübingen, Germany; 8 Department of Urology, KRH Klinikum Siloah, 30449 Hannover, Germany; 9 Department of Urology and Urologic Oncology, Hannover Medical School, 30625 Hannover, Germany

**Keywords:** bladder cancer, urinary biomarkers, nLc4, extracellular vesicles, xCGE-LIF, tumor glycosylation, liquid biopsy, glycosphingolipids, neolactotetraoscylceramide

## Abstract

Glycosphingolipids (GSLs) are promising cancer biomarkers. Using multiplexed capillary gel-electrophoresis with laser-induced fluorescence detection (xCGE-LIF), we profile GSLs in bladder cancer (BC) tissues and find a significant increase in neolactotetraosylceramide (nLc4) compared to matched normal tissue (*n* = 30). Immunofluorescence confirms tumor-specific nLc4 expression in both non-muscle-invasive BC (NMIBC) and muscle-invasive BC (MIBC), colocalizing with luminal and basal urothelial markers. Analysis of paired tissue/urine samples, along with BC cell lines, reveals secretion of nLc4 associated with extracellular vesicles in MIBC. Urine profiling shows elevated nLc4 levels in BC patients (*n* = 16) versus controls (*n* = 50; area under the curve [AUC] 0.75; accuracy 82%). To support clinical translation, we apply an anti-nLc4 ELISA in a discovery cohort (*n* = 18) and a multi-center validation cohort (*n* = 123). In the validation set, urinary nLc4 levels are significantly elevated in MIBC (AUC 0.78; accuracy 64%) and increase with disease severity. These findings support the potential of urinary nLc4 as a non-invasive biomarker for BC detection.

## Highlights

• nLc4 is a tumor-specific glycosphingolipid elevated in bladder cancer tissue
• nLc4 is expressed in both luminal and basal-like bladder tumor cell populations
• nLc4 is detected in urine and enables non-invasive diagnosis of bladder cancer

## Graphical abstract

**Figure:**
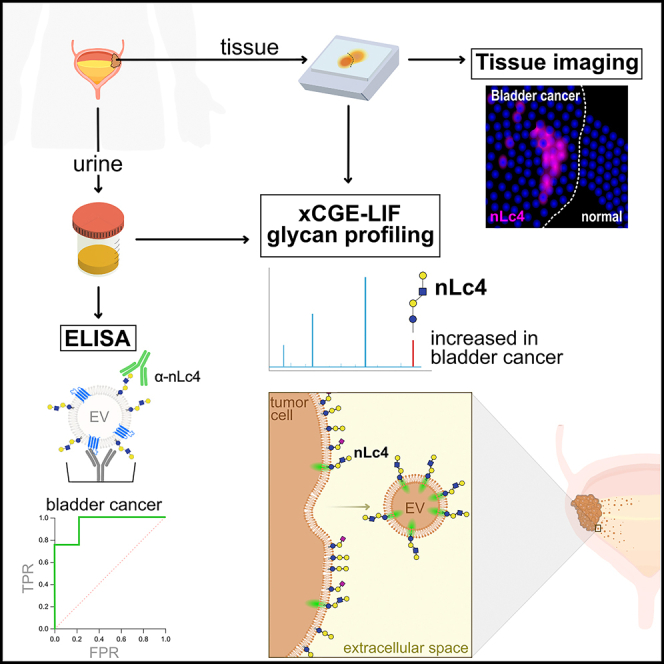

## Teaser

Moreira et al. show that the glycosphingolipid nLc4 is increased in bladder cancer tissue and patients’ urine. nLc4 is selectively expressed in tumor cells and secreted via extracellular vesicles. Its detection by ELISA enables accurate, non-invasive diagnosis and stratification, supporting its potential as a biomarker and therapeutic target.

## Introduction

Bladder cancer (BC) is the most common malignant tumor in the urinary system, with transitional cell carcinoma being its main pathological feature. Worldwide, BC is the 9th most common cancer and accounted for 19.9 million cases and 9.7 million deaths in 2022.^1^ It is more prevalent in men than in women, yet women often present with more advanced disease and experience worse outcomes, reflecting potential biological and clinical disparities.^2^ Currently, the diagnosis of BC is based on cystoscopic examination and histological evaluation of tissue biopsies, usually done in patients already with signs or symptoms of BC. Moreover, patients are monitored by cystoscopy up to four times a year during aftercare. Being an invasive procedure that can cause complications like bleeding or urinary tract infections, cystoscopy is often accompanied by urine cytology, a test that screens for exfoliated tumor cells in the urine. Although representing a simple and non-invasive method, urine cytology lacks adequate sensitivity and overall efficacy to be used as the primary method of histological diagnosis.^3^ Moreover, while more than 30 urinary biomarkers have been recognized so far to diagnose BC, only a few have formal indication for clinical practice.^4^

BC is pathologically classified into non-muscle-invasive (NMIBC) and muscle-invasive (MIBC) forms. NMIBC represents approximately 75% of all newly diagnosed cases and encompasses stages Ta, T1, and carcinoma *in situ* (Tis), which are confined to the mucosa or submucosa. In contrast, MIBC involves invasion into the muscularis propria (stage T2 and beyond) and is associated with a worse prognosis and higher risk of metastasis. Although most NMIBC cases are less aggressive, high-grade (HG) NMIBC tumor such as pTa HG and pT1 HG carry a significant risk of progression to MIBC.^5^

After diagnosis, treatment varies according to cancer type and stage and may include transurethral resection; intravesical therapy; radical cystectomy; and chemo-, radio- and immunotherapy.^6^ These modalities contribute to a significant economic burden and highlight the need for more targeted strategies. Therefore, there is an ongoing effort to identify new reliable biomarkers in urine and tissue, to serve as accessible tools for the early diagnosis and treatment of BC.

Overall, tumor cells display a set of oncogenic-related cellular modifications that confer them selective advantage. Among them, alterations in glycosylation pathways are a common feature of all cancer hallmark abilities, with most Food and Drug Administration (FDA)-approved tumor markers being glycoproteins or glycan antigens.^7^ Glycosphingolipids (GSLs), composed of a hydrophobic ceramide backbone linked to a hydrophilic carbohydrate chain, are major glycolipids in mammals.^8^ These glycans, mainly classified within ganglio-, globo-, or (*neo*)lacto-series, interact with key molecules at the cell membrane level, playing essential roles in mediating cell-cell interactions and modulating signal transduction pathways.^8^^,^^9^ Aberrant expression of specific GSLs is strongly associated with tumor initiation and malignant transformation in many types of human cancer, altering cell growth, adhesion, and motility.^10^ Given the cell-type specificity of GSL expression, BC-specific alterations can be profiled by multiplexed capillary gel-electrophoresis with laser-induced fluorescence detection (xCGE-LIF), a fast, high-throughput-compatible glyco-analytical platform that enables the screening of potential glycan markers derived from complex biological samples.^11^

In this study, we applied xCGE-LIF to profile GSL in primary BC tumors and non-malignant surrounding material, as well as urine samples of BC patients and non-BC individuals. This approach unraveled the global GSL profile of BC cohorts, which led to the identification of neolactotetraosylceramide (nLc4) as a biomarker candidate for BC.

## Results

### GSL analysis of BC tissues uncovered tumor-specific signatures

**Figure 1:**
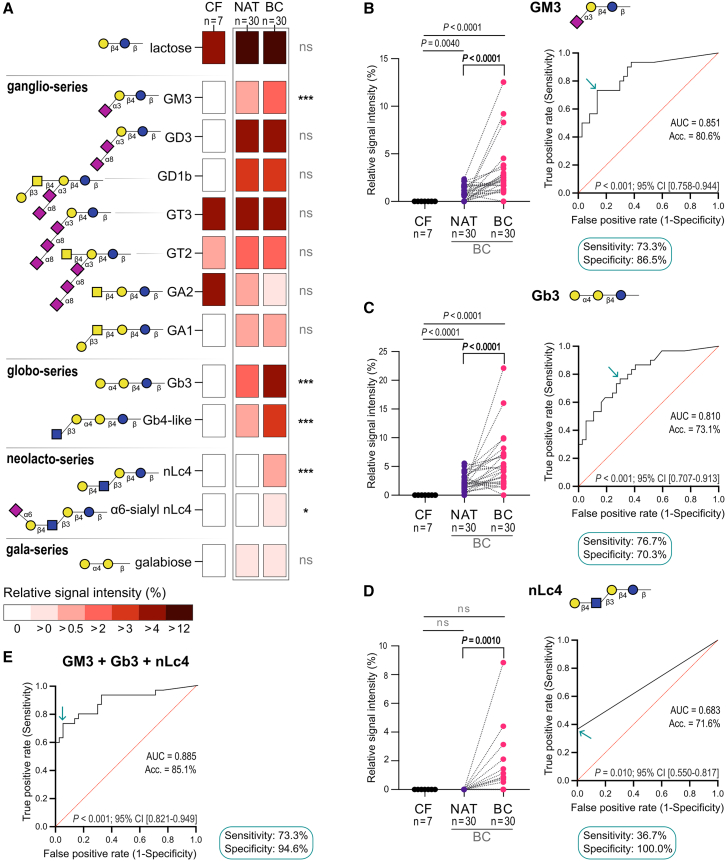
Glycosphingolipid profiling of bladder cancer tissue (A) GSLs were analyzed in bladder cancer (BC, *n* = 30), normal adjacent tissue (NAT, *n* = 30), and cancer-free (CF, *n* = 7) tissue samples using xCGE-LIF. Heatmap shows mean relative signal intensities of 13 GSLs identified in BC. *q* values are from Mann-Whitney tests with false discovery rate (FDR) 5%. ∗*q* < 0.05; ∗∗∗*q* < 0.001; ns, not significant. (B–D) Subset of GSLs overexpressed in BC and undetectable in CF. Levels of (B) GM3, (C) Gb3, and (D) nLc4 are shown for CF and paired NAT/BC samples (left). Mann-Whitney and Wilcoxon tests were used. ROC curves distinguish BC from CF and NAT (right). Exact *p* values are shown; ns, not significant. (E) ROC curve analysis combining GM3, Gb3, and nLc4. AUC, accuracy, *p* values, and 95% confidence interval are shown. Arrows indicate optimal cutoffs for BC detection. See also Figures S1–S5 and Tables S1, S2, and S5.

We quantitatively profiled GSL-derived glycans by xCGE-LIF from formalin-fixed paraffin-embedded (FFPE) tissue samples, detecting 13 different GSL species. Of these, 9 species (GM3, GD3, GD1b, GA1, Gb3, Gb4-like, nLc4, α6-sialyl nLc4, and galabiose) were exclusively found in cancer patients but absent in cancer-free (CF) bladder material (Figure 1A). Particularly, GM3, Gb3, Gb4-like, nLc4, and α6-sialyl nLc4 were statistically significantly increased in BC compared to normal adjacent tissue (NAT). Principal-component analysis (PCA) based on relative GSL abundance demonstrated that CF samples formed a distinct cluster, well separated from BC and—except for one outlier— from NAT samples (Figure S1A). Particularly, GM3 and Gb3 were significantly increased in BC samples compared to NAT (Figures 1B and 1C). nLc4 was not only significantly elevated in BC compared to NAT but also completely absent in all controls (CF and NAT), being exclusively detected in BC samples (Figure 1D). Receiver operating characteristic (ROC) analysis confirmed the diagnostic value of these markers, with GM3 showing the highest area under the curve (AUC: 0.851), followed by Gb3 (AUC: 0.810) and nLc4 (AUC: 0.683). Combining GM3, Gb3, and nLc4 in a multivariate diagnostic model further improved detection accuracy (AUC: 0.885, 85% accuracy, sensitivity: 73.3%, specificity: 94.6%, Figure 1E).

To further explore the molecular basis of GSL-glycosylation alterations in BC, we performed an *in silico* analysis of genes involved in GSL biosynthesis using the publicly available bladder cancer cases from The Cancer Genome Atlas project (TCGA-BLCA).^12^ We found that *A4GALT* (Gb3 synthase) and *ST3GAL5* (GM3 synthase) were significantly downregulated in BC compared to NAT, which does not correlate to our findings by glycan analytics (Figure S5). In contrast, the expression of *B4GALT3* and *B4GALT4*—genes encoding glycosyltransferases involved in the biosynthesis of nLc4—was upregulated in BC. Notably, *ST6GAL1*, which encodes the enzyme that converts nLc4 into α6-sialyl nLc4, was downregulated in tumor tissues, which can further contribute to the accumulation of the nLc4 precursor in BC. Taken all results together, we selected nLc4 as our primary glycan target for further analysis.

### nLc4 is specifically expressed on BC tissue

**Figure 2:**
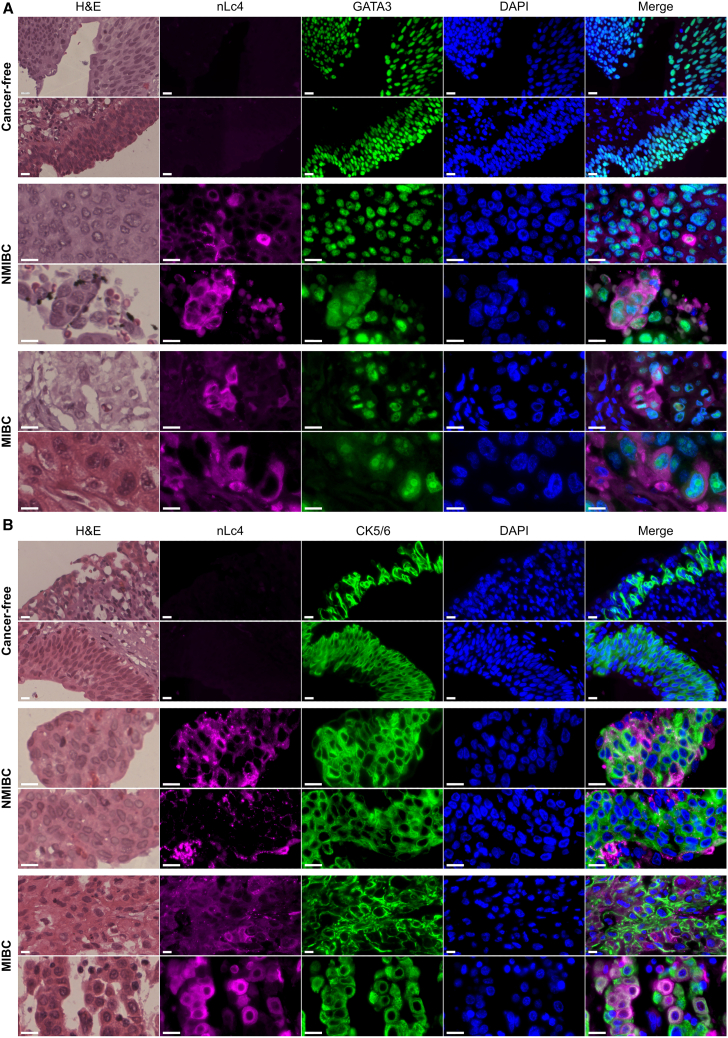
Histological and immunohistochemical analysis of bladder tissues Representative H&E and multiplex immunofluorescence images of cancer-free, NMIBC, and MIBC tissues. Tissues were labeled with anti-nLc4 (clone 1B2, magenta) and either GATA3 (green, luminal marker) (A) or CK5/6 (green, basal marker) (B). Nuclei were visualized with DAPI (blue). Scale bars: 10 μm. A total of 12 biological samples were analyzed; representative images are shown for 2 cancer-free, 2 NMIBC, and 2 MIBC tissues. See also Figures S6 and S7.

To investigate the spatial distribution of nLc4 in bladder tissue, we performed hematoxylin and eosin (H&E) staining and multiplex immunofluorescence (mIF) on FFPE sections of CF, NMIBC, and MIBC tissue samples. Sections were stained with an anti-nLc4 antibody (clone 1B2) in combination with either GATA3, a luminal urothelial marker, or CK5/6, a basal cell marker (Figure 2). H&E staining revealed preserved urothelial architecture in CF tissues, while tumor tissues exhibited marked histopathological alterations, including nuclear atypia and loss of tissue polarity. Immunofluorescence analysis of GATA3 showed nuclear localization in umbrella and intermediate cells of CF tissues, consistent with the organized stratification of healthy bladder mucosa. In contrast, GATA3-positive nuclei were more broadly distributed in NMIBC and MIBC tissues, reflecting disrupted epithelial organization within tumors (Figure 2A). Similarly, CK5/6, which showed membrane-associated staining limited to basal cells in CF urothelium, exhibited a disorganized and widespread distribution in NMIBC and MIBC, indicating an expansion of basal-like features (Figure 2B). Importantly, nLc4 expression was undetectable in CF tissues, while a strong signal was observed in both NMIBC and MIBC tumor regions. In both co-staining panels, nLc4 partially colocalized with GATA3- and CK5/6-positive cells, indicating that nLc4 is expressed by both luminal-like and basal-like tumor cell populations (Figures 2A and 2B).

To confirm the specificity of the anti-nLc4 antibody used in this study, we generated a GSL-deficient BC cell model by knocking out the *UGCG* gene in CAL-29 cells using CRISPR-Cas9. The resulting *UGCG* knockout (KO) cells lacked GSLs but retained surface glycans detected by the *Erythrina* c*ristagalli* lectin (ECL) (Figure S6A). Immunofluorescence and flow cytometry showed strong nLc4 signal in wild-type CAL-29 cells, but not in KO cells, supporting antibody specificity (Figures S6B and S6C). Further validation using BSA-conjugated glycans confirmed that the 1B2 antibody selectively recognized the glycan epitope of nLc4 (Figures S6D–S6F).

To further characterize the molecular identity of nLc4-expressing cells, we analyzed a publicly available single-cell RNA sequencing (scRNA-seq) dataset of urothelial cells isolated from tumor tissue samples, which reflected the BC tumor microenvironment across disease stages (GEO: GSE267718)^13^ (Figure S7). Cells were scored for the expression of glycosyltransferases involved in neolacto-series GSL biosynthesis and putatively classified as nLc4^+^ or nLc4^−^ based on their score (Figures S7C and S7D). Gene set enrichment analysis (GSEA) revealed that nLc4^+^ cells in both NMIBC and MIBC showed upregulation of glycosylation pathways and downregulation of cell cycle and DNA repair programs, compared to nLc4^−^ (Figure S7E). In addition, nLc4^+^ cells in MIBC uniquely exhibited enrichment of transcriptional programs related to epithelial plasticity, metabolic stress, hormonal signaling, and inflammation. These results indicate that although nLc4^+^ cells are present in both tumor stages, in MIBC they display additional features.

### BC cells release extracellular vesicles containing nLc4

**Figure 3:**
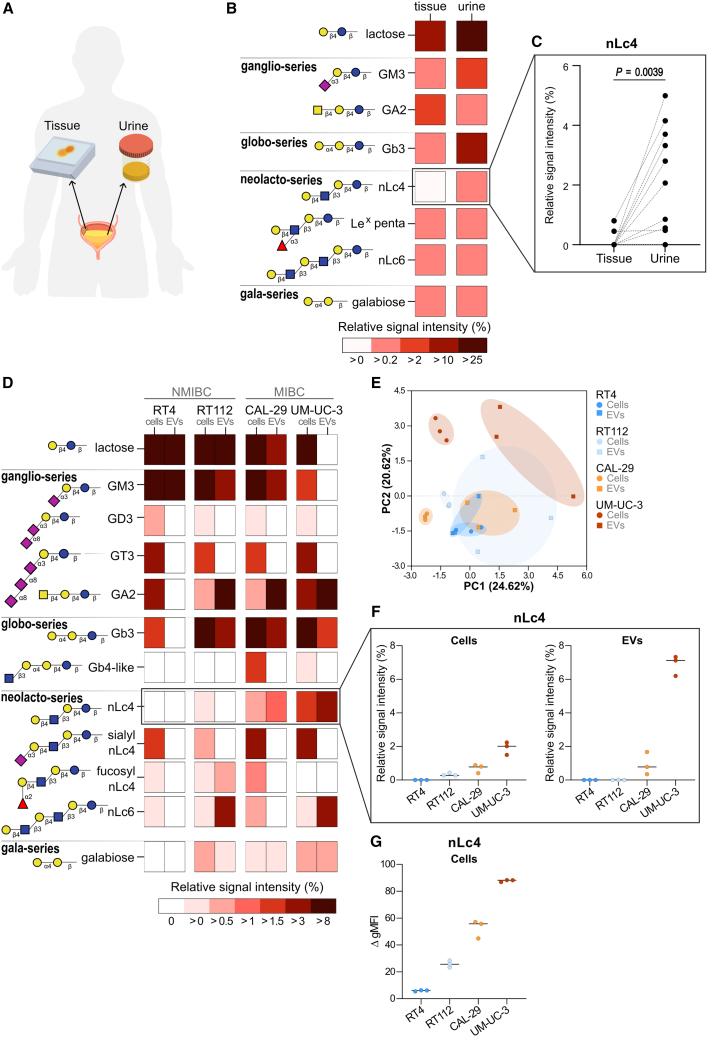
Glycosphingolipid secretion in bladder cancer (A) Schematic of paired tissue and urine sample collection from BC patients (*n* = 13). (B) Heatmap of mean relative signal intensities of GSLs detected in both tissue and urine samples by xCGE-LIF. (C) Paired nLc4 levels in tissue and urine; Wilcoxon test. Exact *p* value is shown. (D) Heatmap of mean relative signal intensities of GSLs detected in four BC cell lines (RT4, RT112, CAL-29, and UM-UC-3) and their EVs by xCGE-LIF. (E) PCA separates cell lines and distinguished cellular vs. EV-derived profiles. (F) Quantification of nLc4 in cells (left) and EVs (right) by xCGE-LIF (*n* = 3). (G) Flow cytometry of nLc4 as Δ geometric mean fluorescence signal relative to negative control (*n* = 3). See also Tables S1, S3, and S5.

To explore whether GSL alterations observed in bladder tumors are also reflected in urine, we performed xCGE-LIF glycan profiling on paired tumor tissue and urine samples (Figure 3A). Relative intensity levels of nLc4 were increased in urine compared to tumor tissue, suggesting that nLc4 may be preferentially secreted or shed by tumor cells (Figures 3B and 3C).

To further investigate this hypothesis, we analyzed four BC cell lines—RT4 and RT122 (both NMIBC), as well as CAL-29 and UM-UC-3 (both MIBC)—using xCGE-LIF to profile GSLs directly from cells and from cell culture supernatant-derived extracellular vesicles (EVs). Each cell line displayed a distinct GSL profile, and principal-component analysis (PCA) analysis showed that cells clustered separately not only from their corresponding EVs (with partial overlap observed in RT4) but also from the cells of the other lines, highlighting the heterogeneity in GSL expression across BC models (Figures 3D and 3E). MIBC cell lines exhibited consistently higher nLc4 expression than NMIBC lines, as confirmed by both xCGE-LIF and flow cytometry (Figures 3F and 3G). Notably, nLc4 was clearly detected in the EV fraction of MIBC cells by xCGE-LIF but not from NMIBC cells. Several other GSL species present in MIBC cells could not be retrieved from the cell culture supernatant (Figure 3D), indicating a selective release of nLc4 into the extracellular space.

### Urinary nLc4 is a non-invasive diagnostic biomarker for BC

Building on the observed enrichment of nLc4 in BC-derived EVs, we next assessed its potential as a non-invasive urinary biomarker. We applied xCGE-LIF analysis to profile GSL-derived glycans from EVs isolated from urine samples of 16 patients with BC and 50 non-BC control individuals. Within the control group, 37 individuals were CF individuals and 13 had other genitourinary or gynecologic cancers (OCs).

**Figure 4:**
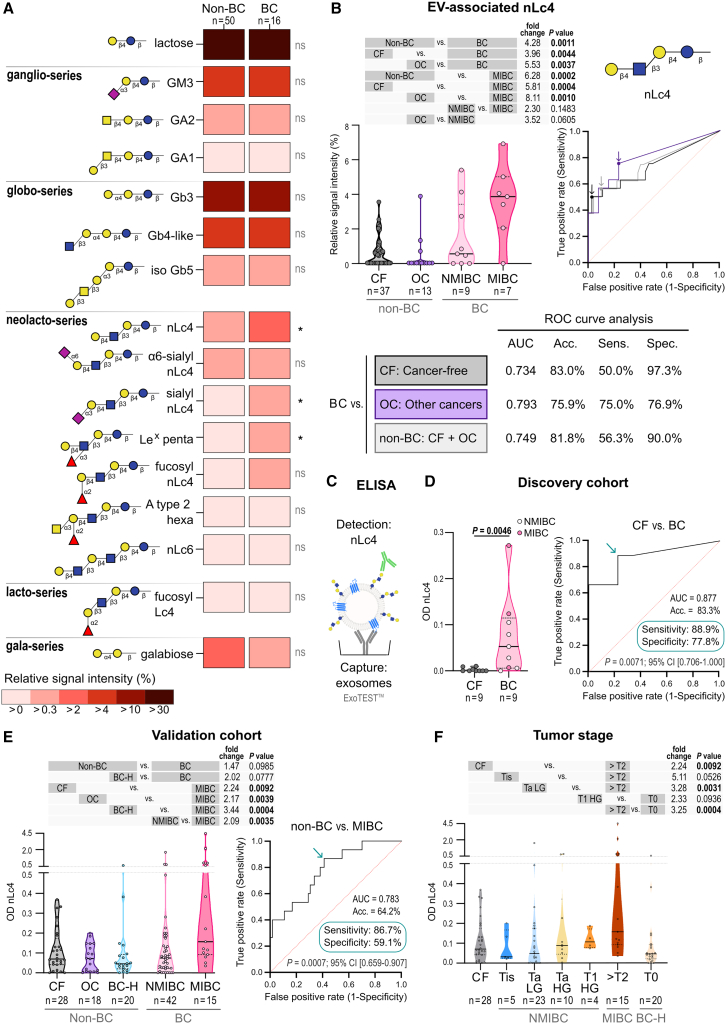
Glycosphingolipid profiling of urinary EVs from bladder cancer patients (A) GSLs were analyzed in bladder cancer (BC, *n* = 16) and non-bladder cancer (non-BC, *n* = 50) urinary EVs using xCGE-LIF. Heatmap shows mean relative signal intensities of 16 GSLs identified. *q* values are from Mann-Whitney tests with false discovery rate (FDR) 5%. ∗*q* < 0.05; ns, not significant. (B) nLc4 levels in CF (*n* = 37), OC (*n* = 13), NMIBC (*n* = 9), and MIBC (*n* = 7) (top left). Mann-Whitney tests were used. Exact *p* values are shown. ROC curves assess diagnostic performance (top right). Arrows indicate optimal cutoffs for BC detection. AUC, accuracy, sensitivity, and specificity are shown (bottom). (C) Schematic of ELISA-based nLc4 detection in urinary exosomes. (D–F) Optical density (OD) levels of nLc4 measured by ELISA. (D) Discovery cohort: CF (*n* = 9) and BC (*n* = 9). (E) Validation cohort: CF (*n* = 28), OC (*n* = 18), BC-H (*n* = 20), NMIBC (*n* = 42), and MIBC (*n* = 15). (F) Stage-specific comparisons among the validation cohort: CF, Tis, Ta LG, Ta HG, T1 HG, ≥T2, and T0. Mann-Whitney tests; only *p* < 0.100 shown. Note: the ExoTEST antibody cocktail was modified between cohort analysis, possibly affecting OD ranges. See also Figures S8–S13 and Tables S1 and S3–S5.

16 GSL species were detected across the analyzed urinary EV samples (Figure 4A). Among these, nLc4, sialyl nLc4, and Le^X^ penta were significantly upregulated in urinary EVs from BC patients compared to non-BC individuals.

Urinary EV-associated nLc4 levels were elevated by a factor of 4.3 in BC patients compared to non-BC individuals (*p* = 0.0011; Figure 4B). Increased levels of nLc4 were consistently observed when comparing all BC patients—or MIBC patients alone—against either CF individuals or patients with other cancers. ROC curve analyses demonstrated that urinary EV-nLc4 discriminated BC from non-BC individuals with an AUC of 0.749, BC from CF with an AUC of 0.734, and BC from OC with an AUC of 0.793.

To facilitate the clinical measurement of nLc4, we adapted a double sandwich ELISA based on urinary exosomes captured using the ExoTEST kit (Figure 4C). This method was applied to two independent patient cohorts. The discovery set included 18 urine samples (9 CF and 9 BC) from the urinary cohort, while the validation set included 123 samples: 28 CF, 18 OC, 20 BC-history (BC-H) patients in remission, 42 NMIBC, and 15 MIBC patients.

In the discovery cohort, urinary exosomal nLc4 levels were significantly higher in BC patients compared to CF individuals (*p* = 0.0046, Figure 4D). ROC curve analysis showed an AUC of 0.877.

In the validation cohort, urinary exosomal nLc4 levels were significantly elevated in MIBC patients compared to CF, OC, BC-H, and NMIBC groups (Figure 4E). ROC analysis showed that nLc4 discriminated MIBC from non-BC controls with an AUC of 0.783. A progressive increase in nLc4 levels was observed with advanced tumor stage, with levels decreasing in patients with tumors in remission (T0: BC-H, Figure 4F). Some comparisons did not reach statistical significance, likely due to small subgroup sizes. To complement traditional analyses and better capture meaningful differences, we applied Cliff’s delta to assess effect sizes across pathological stages (Figure S12C). Moderate to large effect sizes were observed across key transitions, such as between Ta low grade (LG) and T1 high grade (HG), supporting the potential of nLc4 to distinguish between low-risk and aggressive NMIBC subtypes.

Finally, we compared the diagnostic performance of urinary nLc4 levels with BTAstat testing using 60 urine samples from both cohorts (Figure S13A). nLc4 alone achieved the highest overall accuracy (78.7%) and matched the specificity of BTAstat (76.7%) (Figure S13B). Combining nLc4 and BTAstat (positive if either test was positive) increased sensitivity to 90% but reduced specificity to 63.3%, resulting in an overall accuracy of 76.7%.

## Discussion

BC remains a diagnostic challenge, as current gold-standard methods, such as cystoscopy and urinary cytology, are either invasive or lack sufficient sensitivity for early detection.^3^^,^^4^ This highlights the need for robust non-invasive biomarkers.

Multi-omic studies have shown that the genomic, transcriptomic, proteomic, and metabolomic BC molecular profiles allow for the stratification of BC patients into well-defined subtypes, providing a framework for biomarker discovery.^14^^,^^15^ While the glycomic signature is now gaining attention,^16^ the glycolipidomic profiles of bladder carcinomas remain largely unexplored.

Most studies have examined individual GSLs in isolated settings, primarily using tissue samples and cell lines.^10^ Comprehensive characterization of the global GSL landscape, including liquid biopsies, is lacking. In this study, we addressed this gap by using xCGE-LIF, a sensitive platform with high throughput potential,^11^^,^^17^ to profile GSLs across cell lines, primary bladder tumor tissues, and urine samples from BC patients.

Our results demonstrate that BC tissues exhibit a unique GSL signature, distinct from that of CF controls. Specifically, GM3, Gb3, and nLc4 glycans, the core precursors of the ganglio-, globo-, and neolacto-series, respectively, are significantly increased in BC when compared to paired NATs. These findings are consistent with prior studies reporting GSL precursor accumulation in various human tumors as a result of incomplete biosynthesis.^18^^,^^19^ Although previous studies linked GM3 and Gb3 to superficial and invasive BC, respectively,^20^ we did not observe stage-specific differences in our cohort, potentially due to limited sample size.

The behavior of nLc4 was particularly notable. In our cohort, it was consistently upregulated in bladder tumor tissues, while entirely absent from both cancer-free and matched adjacent urothelium. This tumor-restricted expression pattern may be explained by the upregulation of *B4GALT3* and *B4GALT4*, as shown in the TCGA bladder cancer dataset. Our findings provide large-scale evidence that, within the bladder, nLc4 is a tumor-associated GSL with no detectable expression in surrounding normal tissue.

To assess spatial expression of nLc4 in the tumor context, we performed multiplex immunofluorescence staining alongside GATA3 and CK5/6, markers of luminal and basal differentiation, respectively.^21^ These markers showed distinct staining patterns in tumor versus normal bladder mucosa. In contrast, nLc4 expression was entirely restricted to tumor cells in both NMIBC and MIBC, with no signal in any region of normal bladder tissues. Notably, nLc4 colocalized with both GATA3- and CK5/6-positive cells, indicating expression across multiple urothelial phenotypes. These findings support nLc4 as a specific and broadly expressed tumor surface marker, relevant for glycan-targeted therapeutic approaches.^6^

Notably, nLc4+ urothelial cells exhibited distinct transcriptional programs between NMIBC and MIBC, suggesting they may play different roles within the tumor microenvironment. For example, increased transforming growth factor β signaling in MIBC-associated nLc4+ cells, known to promote immune suppression, epithelial-mesenchymal transition, and therapy resistance in advanced BC,^22^ points to a more aggressive, immune-evasive phenotype. Follow-up studies should dissect these distinct programs to reveal their contribution to tumor behavior.

Our hypothesis that nLc4 is selectively released by BC cells into the urine or cell culture supernatant via shedding of EVs aligns with prior reports of GSL shedding from tumor cell membranes, where secreted glycans can modulate immune cell function and contribute to immune escape.^23^ While our data suggest this route, direct quantification of nLc4 abundance on EV membranes versus cellular membranes remains an important direction for future studies. Similar glycan-focused EV analyses have been reported in other tumor types,^24^ and applying such methods to nLc4 will be important in future work, to further clarify the mechanisms underlying its secretion.

Having demonstrated that nLc4 is consistently upregulated in bladder tumor tissues and preferentially secreted by BC cell lines, particularly those derived from MIBC, we next evaluated its utility as a non-invasive biomarker. Using xCGE-LIF, we comprehensively profiled the GSL composition of urinary EVs from BC patients and non-cancer controls. Urinary nLc4 levels were significantly elevated in BC patients—particularly those with MIBC—compared to both CF individuals and patients with other genitourinary malignancies, indicating high disease specificity. This increase was observed in both male and female patients, suggesting that nLc4 may serve as a gender-independent urinary biomarker.

Interestingly, a large proportion of GSLs detected in urinary EVs by xCGE-LIF contained the nLc4 structure, with most members of the neolacto family showing a positive correlation with their shared precursor. In addition to nLc4, the Le^X^ pentasaccharide was significantly elevated in BC patients, consistent with previous reports proposing Le^X^ as a potential urinary biomarker for BC based on immunostaining of exfoliated cells.^25^ Furthermore, sialylated derivatives of nLc4 were elevated in both tissue and urine samples from BC patients. Recent studies have shown that sialylated neolacto-GSLs can sterically hinder human leucocyte antigen class I (HLA-I) interactions and suppress CD8^+^ T cell activation, suggesting a role in tumor immune evasion.^26^ Isolated studies have reported nLc4 presence in ovarian cancer tissues and IGROV1 cells, as well as in pancreatic tumors and the bone marrow of patients with acute myeloid leukemia.^27^^,^^28^^,^^29^ Our findings now establish the presence and diagnostic relevance of urinary nLc4 in BC, reinforcing its potential biological and translational significance.

To enable broader clinical applicability, we next assess nLc4 levels in urinary exosomes using a standard ELISA-based approach, first in a small discovery cohort and subsequently in a larger, well-defined validation cohort. The results ultimately revealed a progressive increase in urinary nLc4 levels across the continuum of disease severity, from carcinoma *in situ* to HG NMIBC and MIBC, with levels decreasing in patients with tumors in remission. While not all comparisons were significant, effect size analysis revealed meaningful differences between clinically relevant subtypes, such as Ta LG and T1 HG tumors. This is clinically relevant, as HG NMIBC lesions are biologically more aggressive and carry a substantially higher risk of progression to muscle-invasive disease.^30^ Early identification of these cases is critical, as affected patients may benefit from intensified treatment strategies, including intravesical bacille Calmette-Guérin (BCG) therapy, closer surveillance, or consideration of early radical cystectomy.^31^ Altogether, the differential expression and secretion of nLc4 between NMIBC and MIBC potentially reflect distinct biological features and clinical requirements of these tumor subtypes, further suggesting its potential role not only as a diagnostic marker but also as a tool for early risk stratification in NMIBC, a current clinical unmet need.^32^

Finally, when benchmarked against BTAstat, a widely used FDA-approved urinary test for BC diagnosis, nLc4 demonstrated superior sensitivity while maintaining comparable specificity. Combining both tests further improved diagnostic performance, particularly by increasing the negative predictive value, a clinically valuable feature for safely excluding disease and minimizing unnecessary invasive procedures such as cystoscopy. The diagnostic accuracy of the nLc4 ELISA supports its potential for clinical implementation, offering a technically feasible promising and cost-effective alternative, or complement, to existing diagnostic tools.

Beyond its diagnostic utility, nLc4’s tumor-restricted expression within the bladder and its cell surface localization suggest it may also hold potential as a therapeutic target. In particular, it could represent a candidate for glycan-directed therapies or serve as a biomarker for predicting response to emerging treatment, such as antibody-drug conjugates and immunotherapy. This is particularly relevant in the context of MIBC, where recent studies highlight the importance of biomarker-guided treatment strategies.^33^^,^^34^

In conclusion, our study provides a comprehensive glycosphingolipidomic profiling of BC tissues and urine, identifying nLc4 as a cancer-specific GSL with distinct diagnostic and biological roles in early versus advanced disease stages. These findings highlight nLc4 as a strong candidate for urinary biomarker development and a potential target for future glycan-directed therapies in BC.

### Limitations of the study

The initial tissue and urinary cohorts used for biomarker discovery were limited in size and not intended for stratification analyses. A gender imbalance in the tissue cohort may have reduced the power to detect gender-specific differences in GSL expression. Although validation was performed in a larger, multi-center cohort, future studies with broader patient populations are needed to confirm the clinical utility of nLc4 in BC. While ELISA-based detection of urinary nLc4 showed promising results and is technically feasible, broader clinical implementation will require rigorous validation and the development of automated, high-throughput workflows. This study was cross-sectional and restricted to a single time point, preventing evaluation of nLc4 dynamics during treatment or follow-up. Longitudinal data will be important to assess its potential for disease monitoring. Lastly, although our data support nLc4 enrichment in EVs, we did not directly compare its abundance on EVs versus cellular membranes; glycan-specific localization methods will be important to confirm selective secretion.

## Resource availability

### Lead contact

Further information and requests for resources and reagents should be directed to the lead contact, Prof. Dr. Falk F.R. Buettner (buettner.falk@mh-hannover.de).

### Materials availability

This study did not generate new unique reagents.

### Data and code availability

• xCGE-LIF GSL profiling data have been deposited in Figshare and are publicly available as of the date of publication at Figshare: 29336381 (https://doi.org/10.6084/m9.figshare.29336381).
• No previously unreported custom computer code or mathematical algorithm was used to generate results central to the conclusions.
• Any additional information required to reanalyze the data reported in this paper is available from the lead contact upon reasonable request.

## Acknowledgments

This study was supported by the 10.13039/501100001659Deutsche Forschungsgemeinschaft (DFG, German Research Foundation) for Forschungsgruppe FOR2953 (project number 409784463 for F.F.R.B.), for Forschungsgruppe FOR2509 (project number 289991887 for F.F.R.B.), and for the Research Training Group GRK2543/2 (for M.L.B.); by the 10.13039/100011937Lower Saxony Ministry of Science and Culture (Niedersächsisches Vorab) for the REBIRTH-Center (for F.F.R.B.); by the German Ministry for Education and Research (10.13039/501100002347BMBF 13GW0399B for J.S. and J.H.B.); and by the 10.13039/100008672Wilhelm Sander-Stiftung (project number 2019.035.01 for J.S. and J.H.B.). We thank the German Study Group of Bladder Cancer (DFBK e.V.), Munich, Germany, and the German Society of Urology, UroFors Consortium (Natural Scientists in Urological Research), Düsseldorf, Germany, for their cooperative support through the membership of M.J.H., M.L.B., and D.S. We would like to thank Prof. Dr. Rita Gerardy-Schahn and Prof. Dr. Christoph Garbers (Institute of Clinical Biochemistry, Hannover Medical School, MHH) for providing an inspiring research atmosphere and general laboratory equipment. We also acknowledge Edda Christians for expert technical assistance, Tom Siol for assistance in photomicrography of tissue stains, Sara Vicente for the support on the graphical design of schematic figures, and Assoc. Prof. Ulla Mandel and Prof. Henrik Clausen for providing the hybridoma expressing the 1B2 antibody detecting nLc4. We acknowledge all the subjects that accepted participating in this study and all the clinical staff involved in sample collection.

## Author contributions

Conceptualization, I.B.M. and F.F.R.B; methodology, I.B.M., C.R., J.K., J.B., and M.M.V.; investigation, I.B.M., C.R., J.K., J.B., M.M.V., J.S., A.O., J.H.B., and F.F.R.B.; data curation, I.B.M. and M.M.V.; formal analysis, I.B.M., M.M.V., and A.G.; resources, J.S., M.J.H., M.E.R.M., D.S., M.L.B., Y.L., J.H.B., H.T., and F.F.R.B.; writing – original draft, I.B.M. and F.F.R.B.; writing – review and editing, I.B.M. and F.F.R.B.; visualization, I.B.M., J.K., M.M.V., and J.S.; project administration, F.F.R.B.; supervision, F.F.R.B.

## Declaration of interests

Patent is granted for F.F.R.B., C.R., A.O., and H.T.: Analytical method and immunological treatment for bladder cancer (EP 20750248.5).

## Declaration of generative AI and AI-assisted technologies in the writing process

During the preparation of this work, the authors used ChatGPT (OpenAI) in order to improve the clarity and readability of the text. After using this tool, the authors reviewed and edited the content as needed and take full responsibility for the content of the publication.

## STAR★Methods

### Key resources table

**Table:** 

REAGENT or RESOURCE	SOURCE	IDENTIFIER
**Antibodies**		

Anti-human nLc4 (1B2 hybridoma)	Prof. Mandel and Prof. Clausen, Copenhagen Center for Glycomics [Young et al.^35^]	N/A
Anti-human GATA3 (EPR16651)	Abcam, UK	Cat# AB199428; RRID:AB_2819013
Anti-human CK5/6 (EP24 & EP67)	Sigma-Aldrich, Germany	Cat# 356R-14-RUO; RRID:AB_3705493
Anti-human GM3 (GMR6)	Tokyo Chemical Industry, Japan	Cat# A2582; RRID:AB_3105836
*Erythrina Cristagalli* Lectin (ECL) biotinylated	VectorLabs, USA	Cat# B-1145; RRID:AB_2336436
Goat anti-mouse IgM Alexa Fluor™ 488	Thermo Fisher Scientific, USA	Cat# A-21042; RRID:AB_2535711
Goat anti-mouse IgM Alexa Fluor™ 555	Thermo Fisher Scientific, USA	Cat# A-21426; RRID:AB_2535847
Goat anti-rabbit IgG Alexa Fluor™ 488	Thermo Fisher Scientific, USA	Cat# A-11008; RRID:AB_143165
Goat anti-rabbit IgG Alexa Fluor™ 555	Thermo Fisher Scientific, USA	Cat# A-21428; RRID:AB_2535849
Goat anti-mouse Ig-HRP	SouthernBiotech, Germany	Cat# 1010-05; RRID:AB_2728714
Goat anti-mouse IgM (H + L) IRDye 800 CW	LI-COR Biosciences, USA	Cat# 926-32280; RRID:AB_2814919

**Biological samples**		

Paired primary tumor and adjacent normal bladder tissue (FFPE)	Hannover Medical School, Germany	N/A
Normal bladder tissue from cancer-free individuals (FFPE)	Hannover Medical School, Germany	N/A
Urine samples from bladder cancer patients and controls	Hannover Medical School, Germany	N/A
Urine samples from bladder cancer patients and controls	Siloah Hospital Hannover, Germany	N/A
Urine samples from bladder cancer patients and controls	Jena University Hospital, Germany	N/A
Urine samples from bladder cancer patients and controls	Eberhard Karls University of Tübingen, Germany	N/A

**Chemicals, peptides, and recombinant proteins**		

Streptavidin Alexa Fluor^TM^ 647	Thermo FIsher Scientific, USA	Cat# S21374
LudgerZyme Ceramide Glycanase (CGase)	Ludger, Abingdon, UK	Cat# LZ-CER-HM-KIT
8-aminopyrene-1,3,6-trisulfonic acid tridosium salt (APTS)	Sigma-Aldrich, Germany	Cat# A7222
Protein blocking solution	Zytomed Systems, Germany	Cat# ZUC007
Fluorescence antibody diluent	Zytomed Systems, Germany	Cat# ZUC025
Fluoromount-GTM mounting medium with DAPI	Thermo Fisher Scientific, USA	Cat# 00-4959-52
Propidium iodide	Sigma-Aldrich, Germany	Cat# P4170
Lactose	Merck, Germany	Cat# 107660
LacNAc (type II)	Elicityl, France	Cat# GLY008
Lacto-*N*-tetraose (Lc4)	Elicityl, France	Cat# GLY010
Lacto-*N*-neotetraose (nLc4)	Elicityl, France	Cat# GLY021
Bovine serum albumin (BSA)	Merck, Germany	Cat# 810037
Sodium cyanoborohydride (NaBH_3_CN)	Merck, Germany	Cat# 818053
Coomassie Blue (Blaue Jonas)	German Research Products, Germany	Cat# GRP1
Ponceau S	Merck, Germany	Cat# 1042380025

**Critical commercial assays**		

ExoTEST™ ELISA Kit	HansaBioMed Life Sciences, Estonia	Cat# HBM-RTK-POF/TU
BTA stat bladder cancer rapid test	HITADO, Germany	Cat# RLT37050601

**Deposited data**		

xCGE-LIF dataset of BC tissue and urine	This paper	https://doi.org/10.6084/m9.figshare.29336381
Bulk RNA-sequencing data for BC tissue	TCGA-BLCA	https://www.cancer.gov/tcga
scRNAseq dataset of BC tissue	Tran et al. 2024^13^	GEO: GSE267718

**Experimental models: Cell lines**		

CAL-29	DSMZ, Germany	ACC 515
*UGCG* KO CAL-29	This study	N/A
RT4	Dr. M.J.Hoffmann	N/A
RT-112	Dr. M.J.Hoffmann	N/A
J82	Dr. M.J.Hoffmann	N/A
UM-UC-3	Dr. M.J.Hoffmann	N/A

**Oligonucleotides**		

*UGCG* FW: 5′-CACCGTTAGGATCTACCCCTTTCAG-3′	This study	N/A
*UGCG* RV: 5′-AAACCTGAAAGGGGTAGATCCTAA-3′	This study	N/A

**Recombinant DNA**		

pCas9_GFP plasmid	Addgene, USA	Plasmid #44719; RRID:Addgene_44719
gRNA_AAVS1-T2 plasmid	Addgene, USA	Plasmid #41818; RRID:Addgene_41818

**Software and algorithms**		

GeneMapper (v.3.7)	Applied Biosystems	https://genemapper.software.informer.com/3.7/
ImageJ	NIH	https://imagej.nih.gov/ij/
GraphPad Prism (v.9)	GraphPad	https://www.graphpad.com/
SPSS v28.0.1.1	IBM Corp.	https://www.ibm.com/
R (v.4.1.3)	R Foundation	https://www.r-project.org/
FlowJo (v.10)	FlowJo	https://www.flowjo.com/
TCGA Biolinks Bioconductor Package (v.2.25.3)	Bioconductor	https://bioconductor.org
DESeq2 Bioconductor Package (v.1.34.0)	Bioconductor	https://bioconductor.org

**Other**		

Chromabond C18 ec polypropylene column	Macherey-Nagel, Germany	Cat# 730012
Precellys 24 tissue homogenizer & lysing kit	Bertin Instruments, France	Cat# P000973-LYSK0-A.0
Ultrafiltration units (MWCO = 30 kDa, Vivaspin 500)	Sartorius Stedim Biotech, Germany	Cat# VS0122
ABI PRISM 3100-Avant Genetic Analyzer	Thermo Fisher Scientific, USA	N/A
Zeiss Observer.Z1 microscope with AxioCam MRm camera	Zeiss, Germany	N/A
Metafer Scanning Platform	MetaSystems Hard & Software, Germany	N/A
VSViewer software	MetaSystems Hard & Software, Germany	N/A
CyFlow® flow cytometer	Partec, Germany	N/A
BioTek Epoch Microplate Spectrophotometer	BioTek Instruments, USA	N/A
LI-COR Odyssey imager	LI-COR Biosciences, USA	Model 9120

### Experimental model and study participant details

#### Human specimens

A total of 30 paired primary tumor and adjacent normal bladder tissue, and 7 normal bladder tissue samples were collected at Hannover Medical School (Germany) and were formalin-fixed paraffin-embedded (FFPE) prior to xCGE-LIF analysis. Additionally, 12 samples were included for mIF analysis (results are shown for 2 cancer-free, 2 NMIBC and 2 MIBC samples). Urine samples were collected from 16 patients and 50 non-BC individuals at Hannover Medical School (Germany). Urine samples used as validation cohort for ELISA were collected from 16 BC patients and 8 non-BC controls from Siloah Hospital (Hannover, Germany), from 23 patients and 39 controls from University Hospital Jena (Jena, Germany), and from 16 patients and 17 controls from University Hospital Tübingen (Tübingen, Germany). Patient specimens were included after written informed consent. The study was conducted in accordance with the Declaration of Helsinki, German law, and institutional ethical guidelines. Ethical approvals were obtained from the Ethics Committees of Hannover Medical School (8619_BO_S_2019 and 10183_BO-K_2022), University Hospital Jena (2024-3499-Material), and University Hospital Tübingen (678/2024BO2). Details on age, gender, and tumor type, stage, and grade are available in Tables S1–S4.

#### Cell culture

Urothelial carcinoma cell lines RT4, RT-112, J82, and UM-UC-3 were kindly provided by Dr. M. J. Hoffmann (Düsseldorf, Germany) and cultured in DMEM High Glucose with stable glutamine and sodium pyruvate (L0103, Biowest), supplemented with 10% fetal bovine serum (FBS). The CAL-29 urothelial carcinoma cell line was obtained from DSMZ (Braunschweig, Germany) and cultured in DMEM (Gibco) supplemented with 10% FBS. *UGCG* KO CAL-20 cells were generated using CRISPR/Cas9. All cells were maintained in humidity incubator at 37°C and 5% CO_2_.

### Method details

#### Sample processing for xCGE-LIF

##### Tissue samples

FFPE tissues were annotated by a pathologist and 5 mm biopsy punches were collected from tumor and normal regions. Deparaffinization was done with xylene, followed by homogenization in chloroform/methanol (1:2, v/v) using a Precellys 24 tissue homogenizer at 6500 rpm for 10 s twice. Homogenates were used for glycolipid extraction.

##### Urine samples

Small EVs were isolated via differential centrifugation as previously described.^36^ Frozen urine samples (20 mL) were thawed, vortexed, and centrifuged at 200 g for 20 min, 2000 g for 20 min, and ultracentrifuged at 12,500 rpm for 20 min. Supernatants were filtered and ultracentrifuged at 34,200 rpm for 70 min. The pellet was washed with PBS and ultracentrifuged at 26,500 rpm for 70 min. Pellets were stored at −20°C for glycolipid extraction.

##### Cell samples

CAL-29, RT4, RT-112, J82 and UM-UC-3 cells were grown to 90% confluency, harvested (3 x 10∧6 cells), pelleted, washed with PBS, and stored at −20°C until glycolipid extraction. Cell medium supernatants were collected and processed using the same method as for urine samples.

#### Glycolipid extraction and xCGE-LIF analysis

Glycolipid extraction followed published protocols (Figure S14).^11^ Deparaffinized tissue homogenates and urinary or cell supernatant derived-EV pellets were resuspended in chloroform/methanol (1:2, v/v), sonicated, and centrifuged. The supernatant was collected and the extraction repeated with chloroform/methanol (2:1) and (1:1). Extracts were purified on Chromabond C_18_ columns and digested with LudgerZyme CGase. Released glycans were labeled with APTS, purified via HILIC-SPE, and analyzed by xCGE-LIF using an ABI PRISM 3100-Avant Genetic Analyzer. Data were processed with GeneMapper Software. At the time of measurement, our in-house GSL reference library included 70 validated standards, which were used to guide glycan structure assignment and peak identification. The structure and details of all the GSL glycans detected through xCGE-LIF in this study are provided in Table S5.

#### Multiplex immunofluorescence (mIF)

FFPE sections underwent antigen retrieval in EDTA buffer (pH 9.0) at 98°C for 30 min. Sections were blocked, incubated overnight with primary antibodies against nLc4 (1:100),^35^ GATA3 (1:300) and CK5/6 (1:100) at 4°C, followed by secondary antibody staining with Alexa Fluor-conjugated antibodies (1:500) for 30 min at room temperature in the dark. Samples were mounted with DAPI-containing medium and imaged. Imaging: Whole Slide images (WSIs) of all stains were digitized at 40x magnification using the Metafer Scanning Platform. Representative snapshots were captured from the WSIs using VSViewer software. Additionally, identical sections were stained with haematoxylin & eosin (H&E) and images of matched stained sections were acquired.

#### Enzyme-linked immunosorbent assay (ELISA)

ELISA was performed using the ExoTEST kit to detect nLc4 on urinary exosomes. Urine samples (100 μL) were added to pre-coated ELISA plates, incubated overnight at 37°C, and stained with anti-nLc4 primary antibody (undiluted) for 2h at 37°C followed by HRP-conjugated secondary antibody (1:2000) for 1h at 37°C. Absorbance was read at 450 nm and 570 nm, with the latter subtracted from the former, following the manufacturer’s instructions.

#### Validation of Anti-nLc4 antibody specificity

##### Generation of *UGCG* KO cells

GSL-deficient CAL-29 cells were generated using CRISPR/Cas9 as previously described, targeting exon 2 of the *UGCG* gene, which encodes UDP-glucose ceramide glycosyltransferase (glucosylceramide synthase). The primers and plasmids used are listed in the key resources table. Transfected cells were sorted by flow cytometry FACS and screened for loss of GM3 expression by flow cytometry and immunofluorescence. Knockout efficiency and mutations were confirmed by Sanger sequencing.

##### Immunofluorescence

Cells were fixed with 4% paraformaldehyde, blocked in 5% BSA, and incubated with undiluted anti-nLc4 antibody (1B2 clone) for 1h at room temperature, followed by Alexa Fluor 488-conjugated secondary antibody (1:500) for 1h at room temperature. Nuclei were counterstained with DAPI. Images were acquired using a Zeiss Observer.Z1 fluorescence microscope.

##### Flow cytometry

Cells were stained with ECL lectin biotinylated (1:1000) or anti-nLc4 antibody (undiluted) for 30 min on ice, followed by Streptavidin Alexa Fluor 647-conjugated (1:500) or Alexa Fluor 488-conjugated secondary antibody (1:500) for 1h at room temperature. Propidium iodide (1:1000) was added prior to acquisition to exclude dead cells. Data were acquired on a CyFlow flow cytometer and analyzed using FlowJo v10 (BD Biosciences).

##### Synthesis of BSA-glycoconjugates

Lactose (Lac), *N*-acetyllactosamine (LacNAc, type II), lacto-*N*-tetraose (Lc4), and lacto-*N*-neotetraose (nLc4) were conjugated to bovine serum albumin (BSA) via reductive amination, as previously described.^37^ Briefly, 1 μmol of glycan was incubated with 270 μg BSA and 400 μg freshly prepared sodium cyanoborohydride (NaBH_3_CN) in 0.2 M borate buffer (pH 9.0) in a total volume of 20 μL for 72h at 50°C. The reaction mixture was buffer-exchanged into TBS (20 mM Tris, 150 mM NaCl, pH 7.5) using ultrafiltration (MWCO 30 kDa), and stored at −80°C at 1 mg/mL.

##### Western blot

BSA-glycoconjugates (1 μg) were separated on a 12% SDS-PAGE gel and either stained with Coomassie Blue or transferred onto a nitrocellulose membrane. Membranes were blocked in 5% BSA/PBS for 1h at room temperature and incubated overnight at 4°C with undiluted anti-nLc4 antibody. After washing (PBS +0.02% Tween 20), membranes were incubated with fluorescently labeled anti-mouse IgM secondary antibody (1:10,000) for 1h at room temperature. Signal was detected using the LI-COR Odyssey imaging system.

##### Dot blot

BSA-glycoconjugates were spotted onto nitrocellulose membranes at decreasing concentrations (2 μg, 1 μg, 0.5 μg; 2 μL per spot). After drying, membranes were stained with Ponceau S (0.1% in 5% acetic acid), destained with 0.05 M NaOH, and processed for anti-nLc4 detection as described for Western blotting.

#### TCGA-BLCA data analysis

The transcriptomic data of the BLCA cohort from The Cancer Genome Atlas project was downloaded using the TCGA biolinks Bioconductor package (v2.25.3). Prior to analysis, preprocessing steps were done, data was normalized and differential gene expression between BC and NAT samples was done using the package DEseq2 (v1.34.0).

#### Analysis of publicly available scRNA-seq data

Single-cell RNA sequencing (scRNAseq) data (barcodes.tsv, genes.tsv, and matrix.mtx files) were obtained from the Gene Expression Omnibus (GEO) under accession number GSE267718.^13^ Only samples derived from tumor tissues were included in the analysis (data from patient with ID 2, 3, 4, 5, 6, 7A, 8 and 9). Individual Seurat objects were created for each patient using the Seurat package (v5.2.1), and standard quality control measures were applied. Specifically, genes expressed in less than 5 cells were discarded. Cells expressing more than 200 and fewer than 9,000 genes, that had mitochondrial gene content less than 15% were retained. Each sample was independently processed by normalizing gene expression using the *NormalizeData* function and identifying highly variable genes via *FindVariableFeatures*. For integration, the top variable features were selected using *SelectIntegrationFeatures*, and integration anchors were identified with *FindIntegrationAnchors*. These anchors were then used to generate an integrated dataset using *IntegrateData*. The integrated dataset underwent scaling (*ScaleData*), dimensionality reduction with principal component analysis (*RunPCA*), and embedding into a two-dimensional space using uniform manifold approximation and projection (UMAP) via *RunUMAP*. Cell clustering was performed using FindClusters with a resolution of 0.5, and visualized with UMAP. Cell type annotation of each cluster was performed by comparing gene expression profiles with known marker genes provided in the original study.^13^ Urothelial cells were further analyzed by scoring the potential of nLc4 synthesis, given by the expression of *UGCG*, *B4GALT1-6*, and *B3GNT5*, using the UCell package. Urothelial cells were categorized as “nLc4^+^” or “nLc4^-^” based on whether their UCell scores were above or below the median threshold (0.008), respectively. To assess pathway-level differences between these two urothelial subpopulations, gene set enrichment analysis (GSEA) was conducted using the C2 (curated gene sets) collection from the Molecular Signatures Database (MSigDB).^38^ Visualizations were generated using standard Seurat plotting functions.

### Quantification and statistical analysis

Statistical analyses were conducted using GraphPad Prism (v9), SPSS (v28.0.1.1), and R (v4.1.3). Differences between groups were tested using Multiple Mann-Whitney tests with a 5% FDR, two-tailed unpaired Mann-Whitney tests, or Wilcoxon matched-pairs signed rank tests. ROC analyses were performed to assess diagnostic performance, with AUC, sensitivity, specificity, and 95% CI calculated. Multivariable logistic regression was used for combined GSL marker analysis. Principal component analysis was used to visualize clustering patterns. Correlations between GSL levels were assessed using Spearman’s rank correlation coefficient. Effect sizes for non-parametric comparisons were estimated using Cliff’s delta, a distribution-free measure of effect size suitable for ordinal or non-normally distributed data. *p* values smaller than 5% are statistically significant and interpreted as a difference between the compared groups.

## Supplemental information

Document S1. Figures S1–S14 and Tables S1–S5

Document S2. Article plus supplemental information
