# Supplementary material for: Neolactotetraosylceramide enables urinary detection of bladder cancer
Source: Cell Rep Med. 2025 Jul 23;6(8):102246. doi: 10.1016/j.xcrm.2025.102246 (PMC12432384; doi:10.1016/j.xcrm.2025.102246)
Supplement: Document S1. Figures S1–S14 and Tables S1–S5 [file mmc1.pdf]

Cell Reports Medicine, Volume *6*

Supplemental information

Neolactotetraosylceramide enables urinary detection of bladder cancer

Inês B. Moreira, Charlotte Rossdam, Jonas Kaynert, Julia Beimdiek, Manuel M. Vicente, Jessica Schmitz, Anika Großhennig, Astrid Oberbeck, Michèle J. Hoffmann, Michele E. Rosero Moreno, Daniel Steinbach, Maria L. Barcena, Yannick Lippka, Jan H. Bräsen, Hossein Tezval, and Falk F.R. Buettner

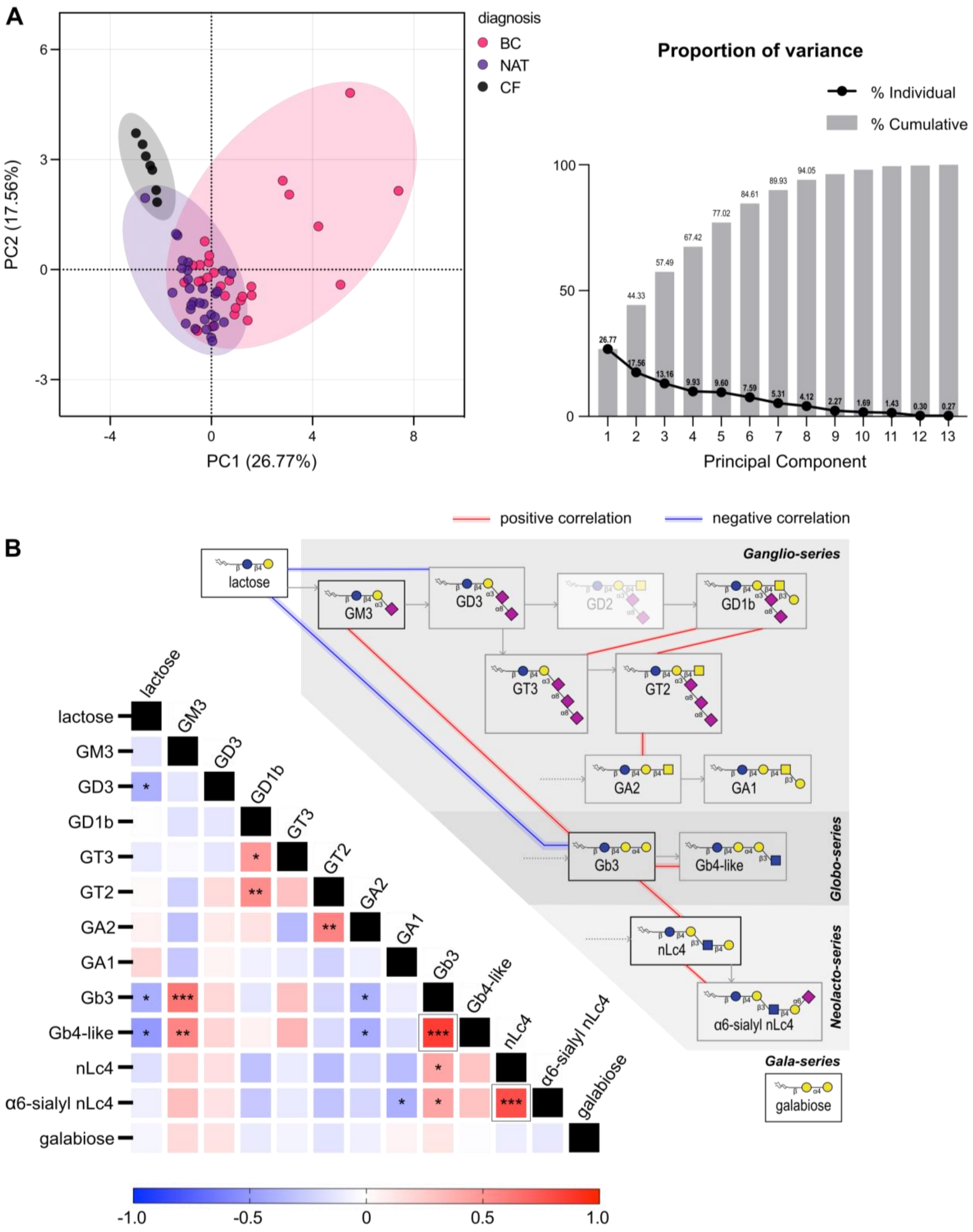

Figure S1. Analysis of tumor associated glycosphingolipids identified in bladder cancer tissue samples. Related do Figure 1.

(A) Principal component analysis (PCA) model based on the relative abundance (%) of individual GSLs expressed in bladder tissues. Separation between cancer, normal adjacent tissue and cancer-free is illustrated on the left; Proportion of variance of the principal components is shown on the right. The top two principal components (PC1 and PC2) explain 44.3 % of the variation within the data.

(B) Correlation matrix of GSL signatures using Spearman correlation coefficients for bladder cancer samples (left). Black boxes highlight the positive correlation between Gb4-like/Gb3 and α6-sialyl nLc4/nLc4; Representation of the GSL biosynthesis pathway and the main correlation relationships are shown on the right. *, *p* value <0.05; **, *p* value <0.005; ***, *p* value <0.001; Blue circle: glucose, yellow circle: galactose, blue square: *N*-acetylglucosamine, yellow square: *N*-acetylgalactosamine, purple diamond: *N*-acetylneuraminic acid.

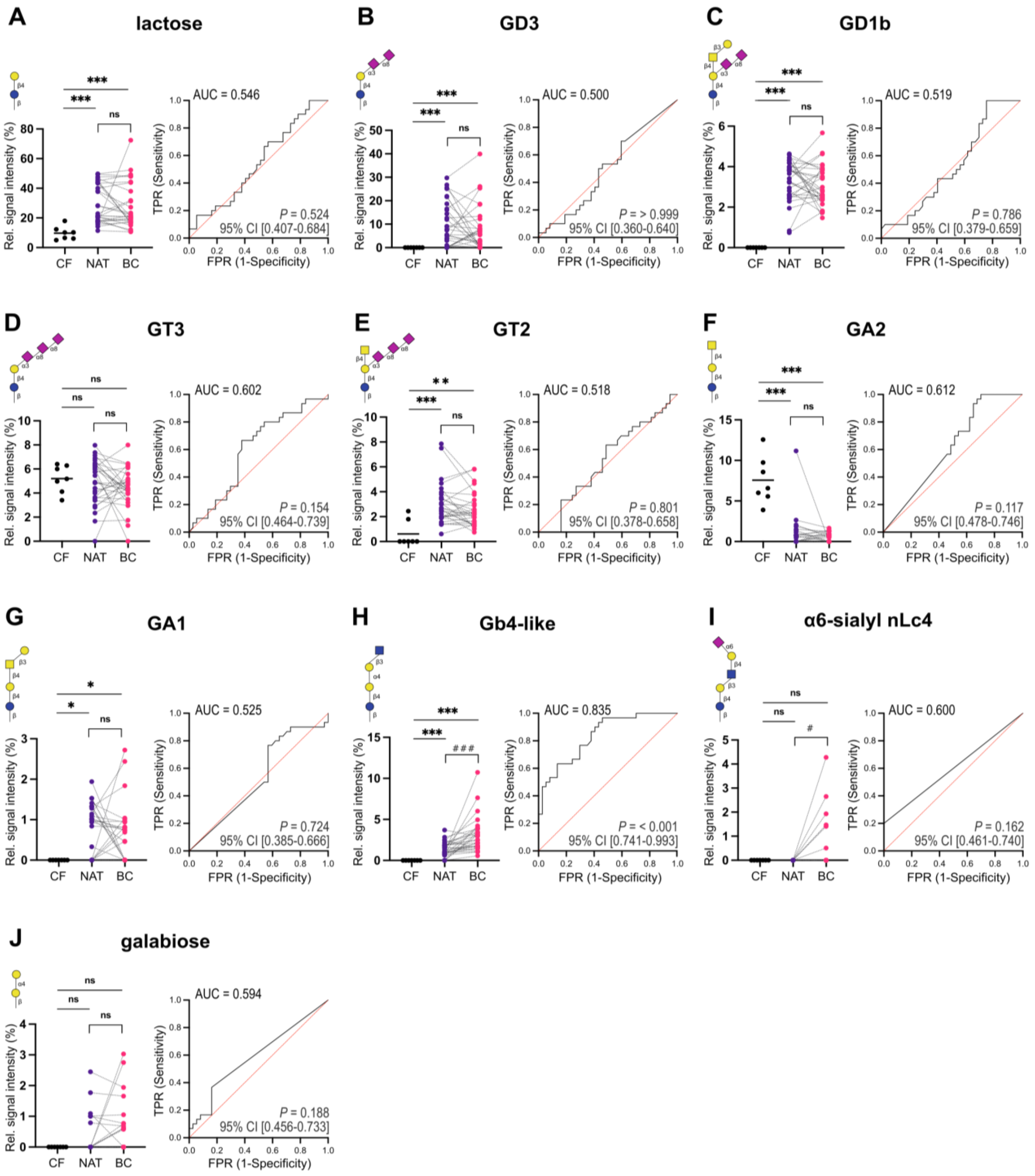

Figure S2. Glycosphingolipid profiling of bladder cancer tissue. Related to Figure 1.
Glycosphingolipids detected in bladder cancer (BC, *n* = 30), normal adjacent tissue (NAT, *n* = 30) and cancer-free (CF, *n* = 7) tissue samples through xCGE-LIF. Relative signal intensity levels of (A) lactose, (B) GD3, (C) GD1b, (D) GT3, (E) GT2, (F) GA2, (G) GA1, (H) Gb4-like, (I) α6-sialyl nLc4 and (J) galabiose in CF and paired analysis between NAT and BC (left). *p* values were calculated using two-tailed unpaired Mann-Whitney test (for comparisons with the CF group) or two-tailed Wilcoxon matched-pairs signed rank test (for NAT vs. BC comparison); ROC curve analysis in bladder cancer detection obtained by calculating the sensitivity and specificity of the test (for CF+NAT vs. BC) at every possible cut-off point and plotting the sensitivity against 1-specificity (right). AUC, *p* value and 95% CI values are shown. * or $^{\#}$, *p* value <0.05; **, *p* value <0.005; ***or $^{\#\#\#}$, *p* value <0.001; ns, non-significant; AUC, area under the curve; CI, confidence interval; TPR, true positive rate; FPR, False positive rate; blue circle: glucose, yellow circle: galactose, blue square: *N*-acetylglucosamine, yellow square: *N*-acetylgalactosamine, purple diamond: *N*-acetylneuraminic acid.

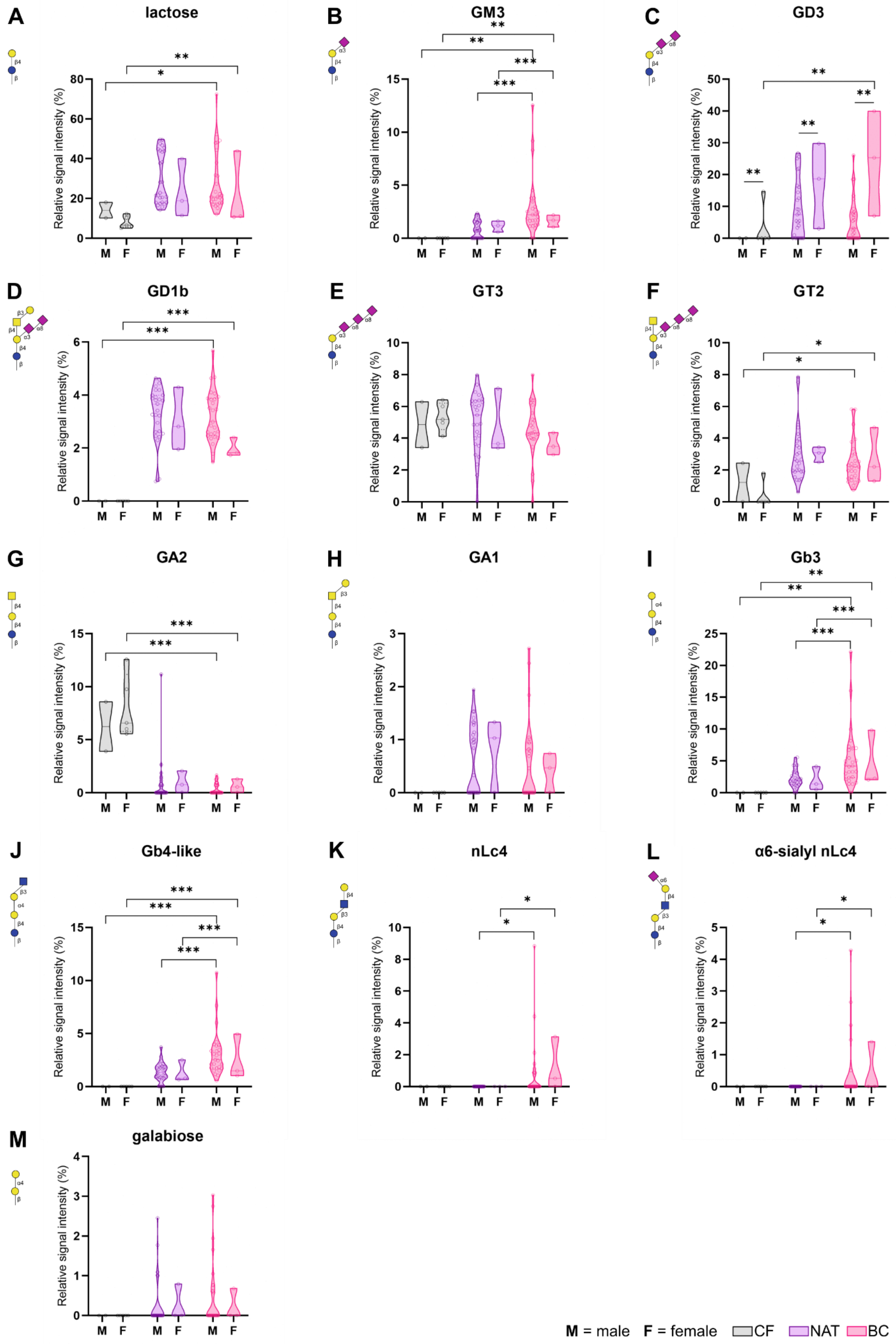

Figure S3. Glycosphingolipid profiling of bladder cancer tissue across gender. Related to Figure 1.

Glycosphingolipids detected in bladder cancer (BC; males, $n = 27$; females $n = 3$), normal adjacent tissue (NAT; males, $n = 27$; females, $n = 3$) and cancer-free (CF; males, $n = 2$; females, $n = 5$) tissue samples through xCGE-LIF. Violin plots reporting the relative signal intensity of (A) lactose, (B) GM3, (C) GD3, (D) GD1b, (E) GT3, (F) GT2, (G) GA2, (H) GA1, (I) Gb3, (J) Gb4-like, (K) nLc4, (L) α6-sialyl nLc4 and (M) galabiose for male (left) and women (right) participant. *p* values were calculated using a 2way ANOVA test. Only statistically significant differences are represented. *, p value <0.05; **, p value <0.005; ***, p value <0.001; blue circle: glucose, yellow circle: galactose, blue square: *N*-acetylglucosamine, yellow square: *N*-acetylgalactosamine, purple diamond: *N*-acetylneuraminic acid.

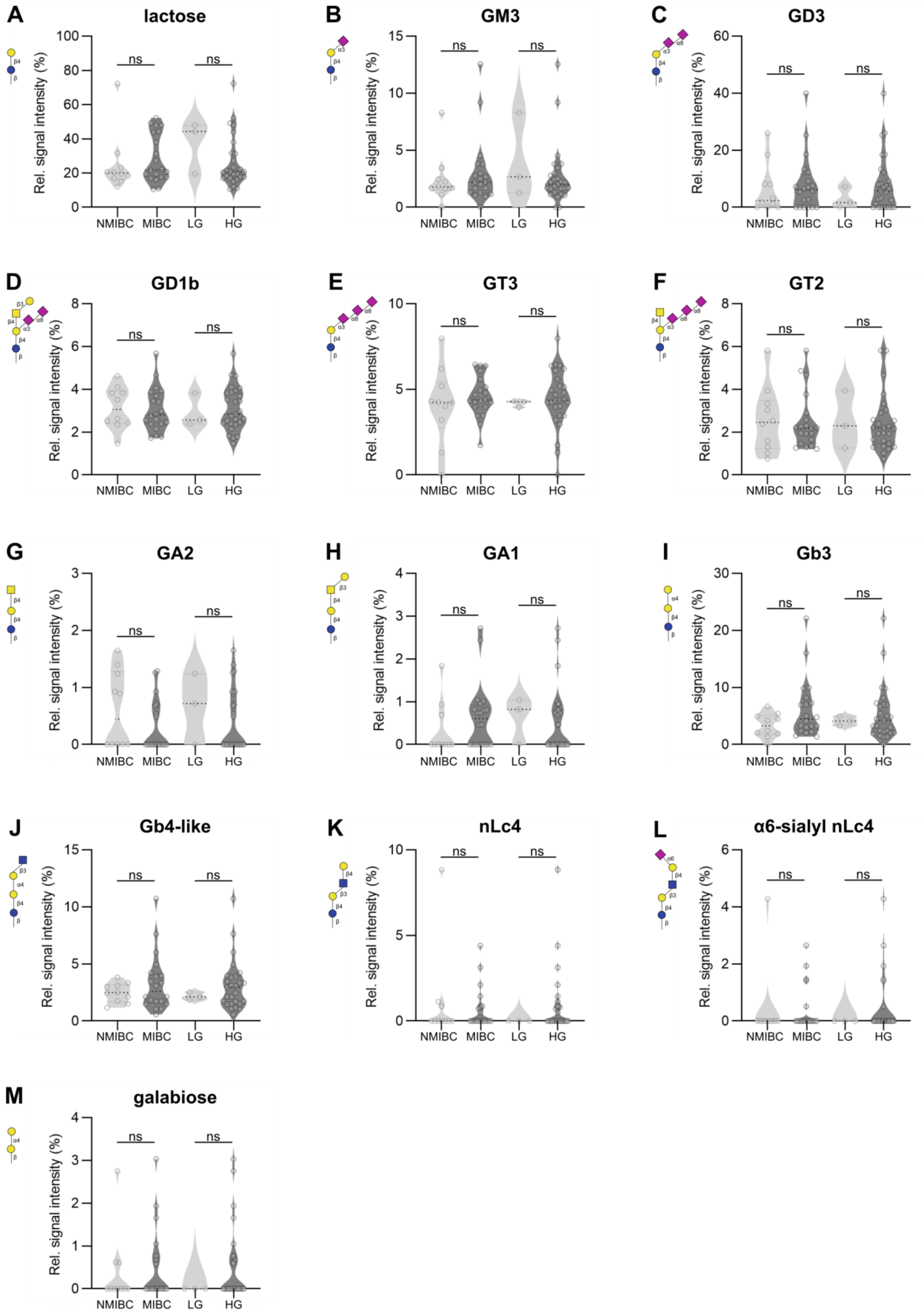

Figure S4. Glycosphingolipid profiling of bladder cancer tissues in regard to tumor classification as NMIBC and MIBC and tumor grade. Related to Figure 1.

Glycosphingolipids detected in bladder cancer (BC, *n* = 30) tissue samples through xCGE-LIF. Relative signal intensity levels of (A) lactose, (B) GM3, (C) GD3, (D) GD1b, (E) GT3, (F) GT2, (G) GA2, (H) GA1, (I) Gb3, (J) Gb4-like, (K)

nLc4, (L) α6-sialyl nLc4 and (M) galabiose across tumor pathological classification (left, NMIBC vs. MIBC) and disease grade (right, LG vs. HG). *p* values were calculated using two-tailed unpaired Mann-Whitney tests. NMIBC, non-muscle invasive bladder cancer (n = 10); MIBC, muscle invasive bladder cancer (n = 20); LG, low grade (n = 3); HG, high grade (n = 27); ns, non-significant blue circle: glucose, yellow circle: galactose, blue square: *N*-acetylglucosamine, yellow square: *N*-acetylgalactosamine, purple diamond: *N*-acetylneuraminic acid.

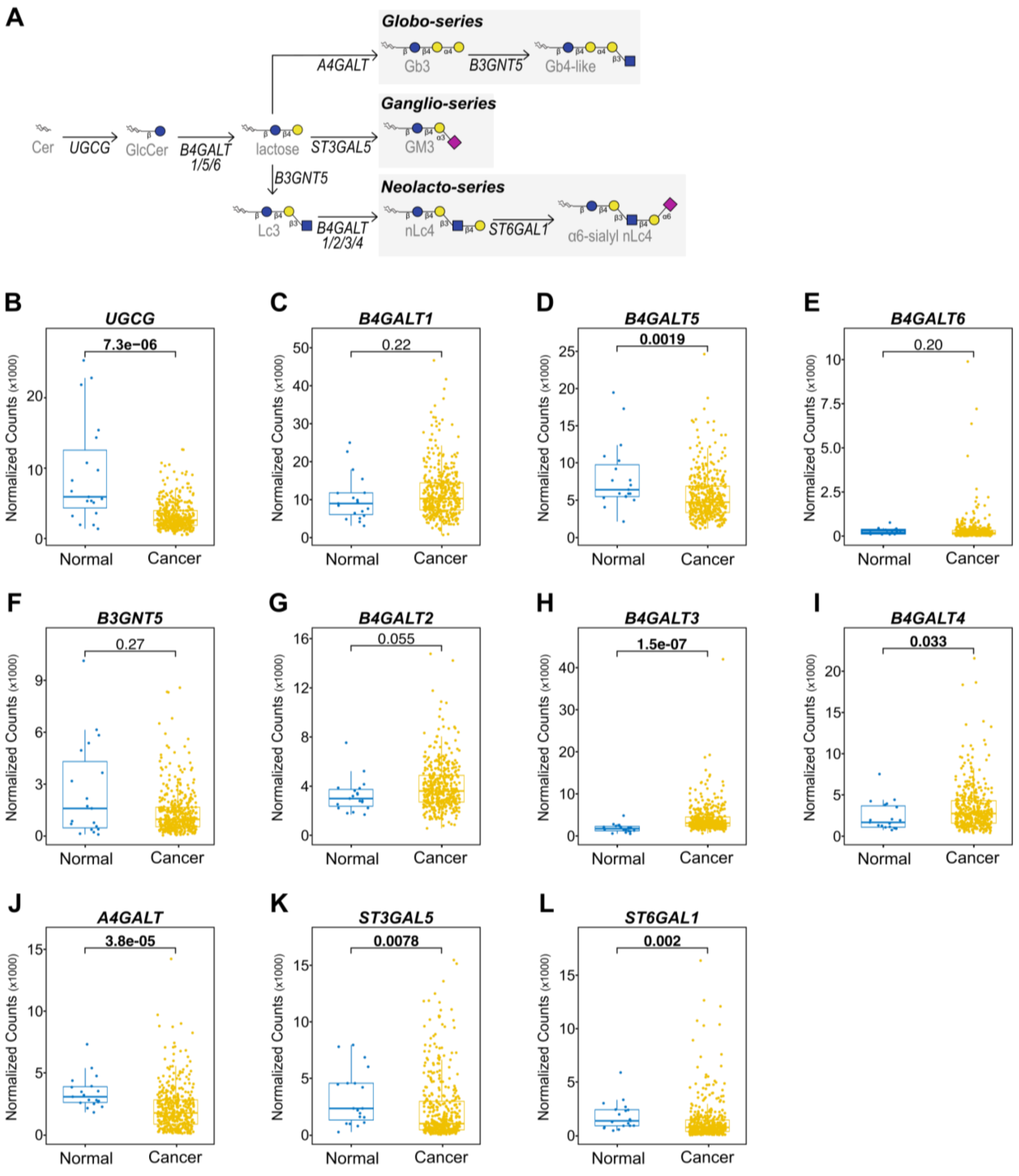

Figure S5. Comparison of gene expression of glycosyltransferases in bladder cancer and normal adjacent bladder. Related to Figure 1.

(A) Biosynthetic pathways of major GSL-series. (B-L) Differences in gene expression in cancer compared to normal surrounding bladder tissue are observed for (B) *UGCG*, (C) *B4GALT1*, (D) *B4GALT5*, (E) *B4GALT6*, (F) *B3GNT5*, (G) *B4GALT2*, (H) *B4GALT3*, (I) *B4GALT4*, (J) *A4GALT*, (K) *ST3GAL5* and (L) *ST6GAL1* glycogenes. Raw data was obtained from the TCGA-BLCA (The Cancer Genome Atlas Urothelial Bladder Carcinoma) dataset. Blue circle: glucose, yellow circle: galactose, blue square: *N*-acetylglucosamine, yellow square: *N*-acetylgalactosamine, purple diamond: *N*-acetylneuraminic acid.

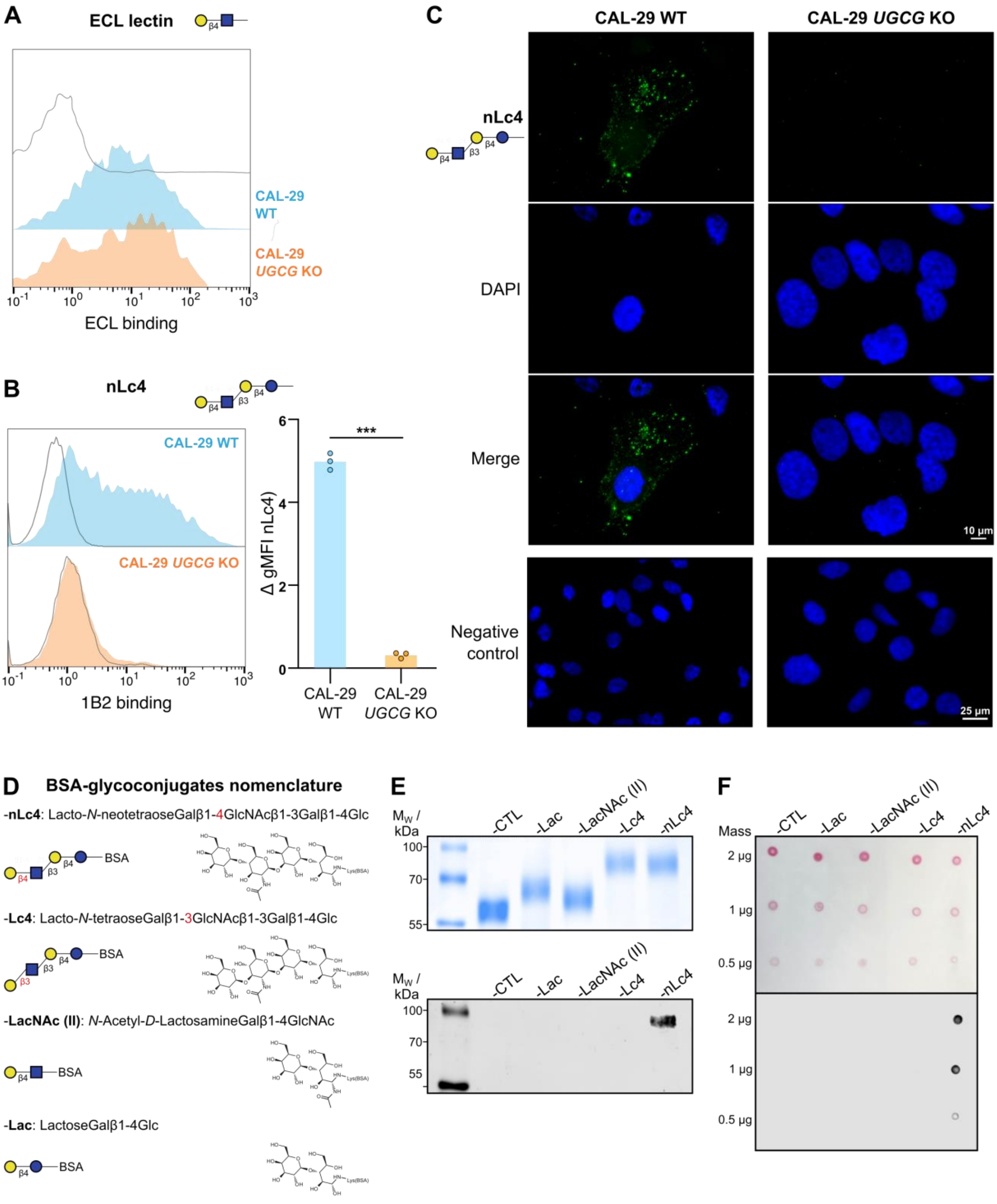

Figure S6. Validation of nLc4-specific antibody. Related to Figure 2.
(A-C) Validation of the anti-nlc4 antibody (1B2 clone) towards GSL detection in BC cells.
(A) Representative flow cytometry histograms showing ECL binding in CAL-29 WT and *UGCG* KO cells. Gray line represents negative control.
(B) Representative flow cytometry histograms showing surface nLc4 signal in CAL-29 WT and *UGCG* KO cells (left). Gray lines represent negative controls. Quantification of nLc4 surface expression as Δ geometric mean fluorescence intensity (ΔgMFI) relative to negative control ($n = 3$) (right). Data were analyzed using unpaired Welch's t test; ***, $P < 0.001$.
(C) Immunofluorescence staining of nLc4 in CAL-29 WT and GSL-deficient CAL-29 *UGCG* KO cells. Scale bars: 10 µm (top), 25 µm (negative control).
(D-F) Validation of 1B2 antibody specificity toward nLc4 using BSA-conjugated glycans.

(D) Nomenclature, Symbol Nomenclature of Glycans (SNFG), and chemical structures of four BSA-glycoconjugates used: Lac (lactose), LacNAc (II), Lc4 and nLc4.
(E) SDS-PAGE (Coomassie stain, top) and Western blot (bottom) of the BSA-glycoconjugates probed with the anti-nLc4 antibody.
(F) Dot blot showing Ponceau S staining (top) and antibody-specific detection of BSA-glycoconjugates (bottom). BSA-glycoconjugates were spotted at increasing concentrations (0.5-2 μg) on nitrocellulose and probed with the anti-nLc4 antibody.
Blue circle: glucose, yellow circle: galactose, blue square: *N*-acetylglucosamine, yellow square: *N*-acetylgalactosamine.

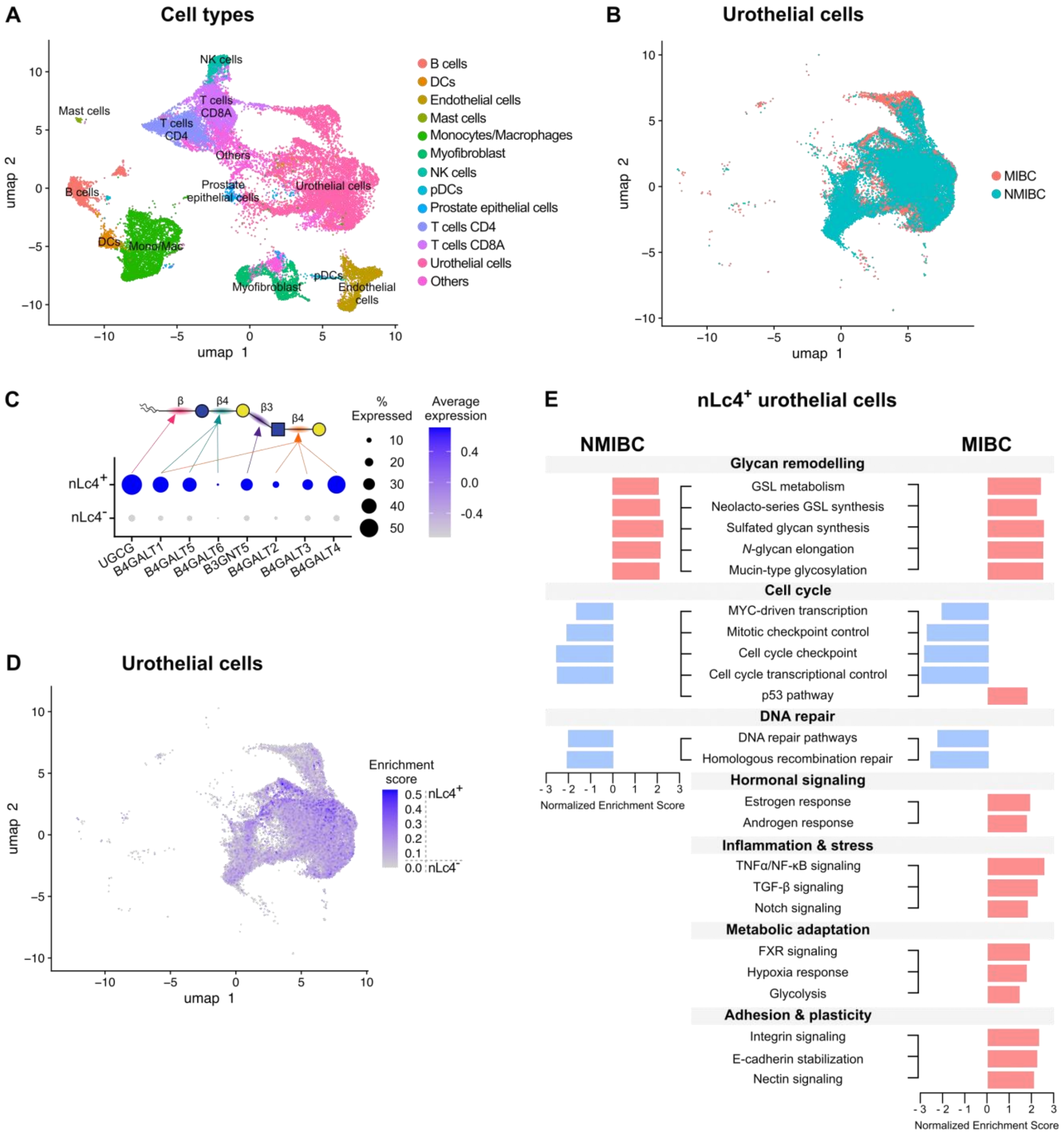

Figure S7. Single cell transcriptomic analysis of nLc4 positive urothelial cells in NMIBC and MIBC. Related to Figure 2.

(A) UMAP visualization of all cells, annotated by cell type.
(B) UMAP of urothelial cells only, colored by tumor stage as NMIBC and MIBC.
(C) Dot plot showing average expression and percentage of cells expressing nLc4 biosynthesis genes (glycosyltransferases) in nLc4^{+} vs. nLc4^{-} urothelial cells.
(D) UMAP of urothelial cells, colored by nLc4 enrichment score (purple scale). Urothelial cells were considered nLc4^{-} if their enrichment score was below 0.008 (median enrichment score across all urothelial cells).
(E) Gene set enrichment analysis (GSEA) of nLc4^{+} urothelial cells comparing NMIBC and MIBC. Normalized enrichment scores (NES) are shown for selected pathways. All gene sets shown have an adjusted *p*-value < 0.05. Red bars indicate upregulated pathways; blue bars indicate downregulation.
scRNA-seq data were obtained from the gene Expression Omnibus (GEO) under accession number GSE267718. DCs, dendritic cells; pDCs, plasmocytoid dendritic cells; NK cells, natural killer cells. Blue circle: glucose, yellow circle: galactose, blue square: *N*-acetylglucosamine, yellow square: *N*-acetylgalactosamine.

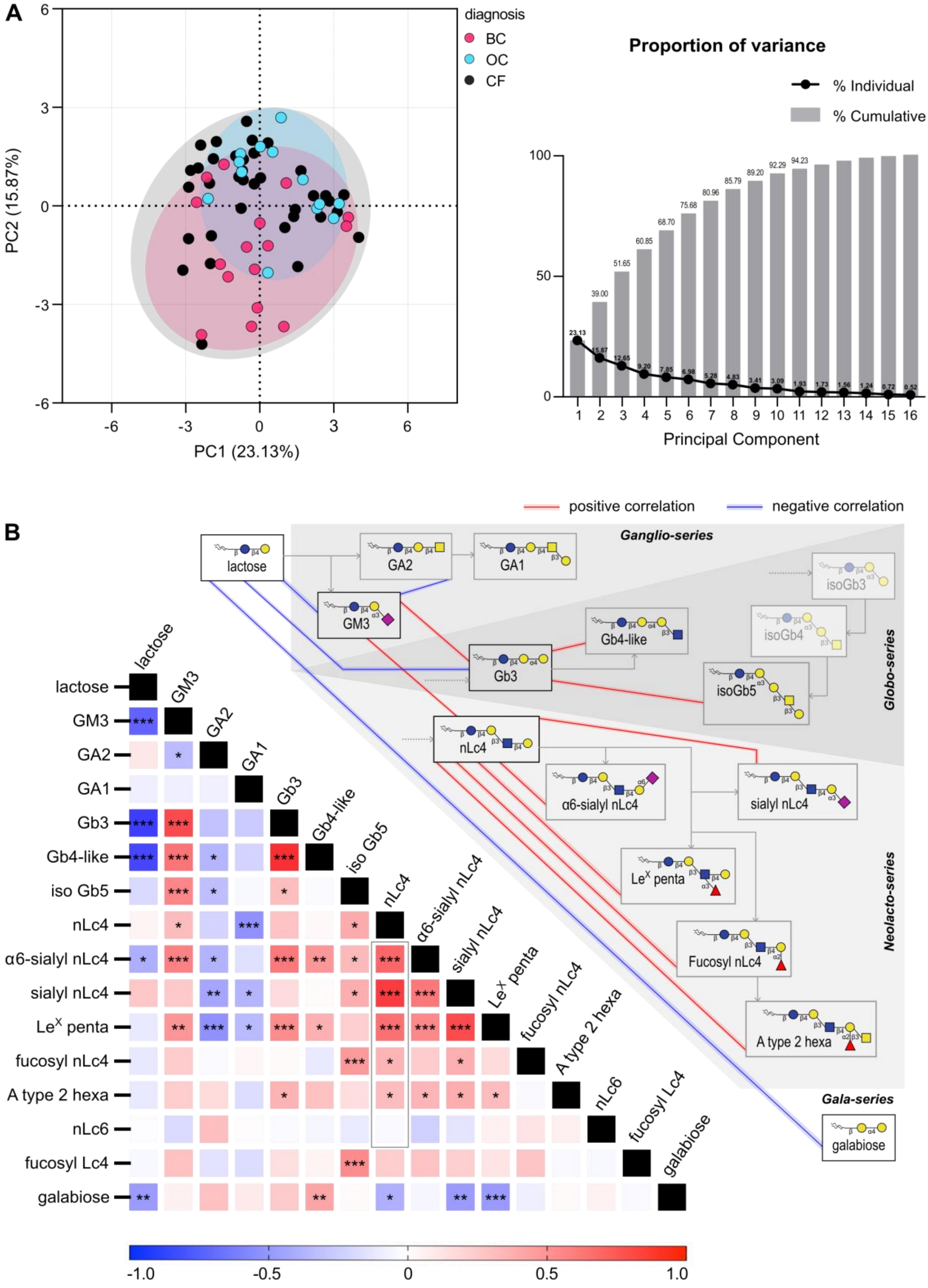

Figure S8. Analysis of tumor associated glycosphingolipids identified in bladder cancer urine samples. Related to Figure 4.

(A) Principal component analysis (PCA) model based on the relative abundance (%) of individual GSLs expressed in urine samples. Separation between bladder cancer, other cancers and cancer-free is illustrated on the left; Proportion of variance of the principal components is shown on the right. The top two principal components (PC1 and PC2) explain 39.0 % of the variation within the data.

(B) Correlation matrix of GSL signatures using Spearman correlation coefficients for bladder cancer samples (left). The black box highlights the correlation between nLc4 and other GSLs of the neolacto-series; Representation of the GSL

biosynthesis pathway and the main correlation relationships are shown on the right. *, *p* value <0.05; **, *p* value <0.005; ***, *p* value <0.001; Blue circle: glucose, yellow circle: galactose, blue square: *N*-acetylglucosamine, yellow square: *N*-acetylgalactosamine, purple diamond: *N*-acetylneuraminic acid, red triangle: fucose. Related to Figure 3.

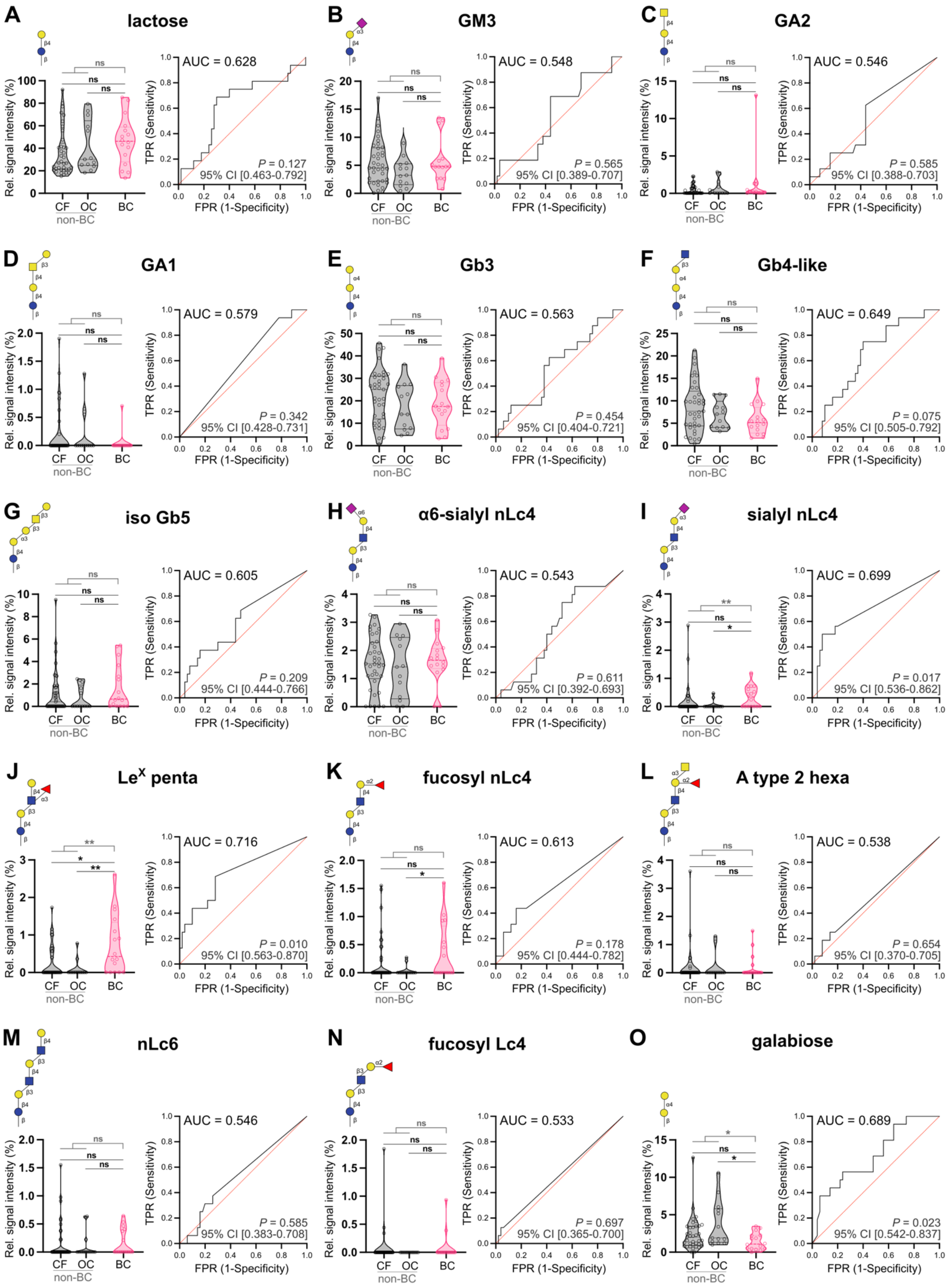

Figure S9. Glycosphingolipid profiling of urinary extracellular vesicles from bladder cancer patients. Related to Figure 4.

Glycosphingolipids detected in urine of patients with bladder cancer (BC; *n* = 16) and without bladder cancer [non-bladder cancer (Non-BC; *n* = 50), comprising cancer-free individuals (CF, *n* = 37) and patients with other cancers (OC, *n* = 13)] through xCGE-LIF. Violin plots reporting the relative signal intensity of (A) lactose, (B) GM3, (C) GA2, (D) GA1, (E) Gb3, (F) Gb4-like, (G) iso Gb5, (H) α6-sialyl nLc4, (I) sialyl nLc4, (J) Le^X penta, (K) fucosyl nLc4, (L) A type

2 hexa, (M) nLc6, (N) fucosyl Lc4 and (O) galabiose (left). *p* values were calculated using two-tailed unpaired Mann-Whitney test; ROC curve analyses of urinary GSLs in patients with BC compared to non-BC individuals as predictive models for BC diagnosis (right). AUC, *p* values and 95% CI values are shown. *, p value <0.05; **, p value <0.005; ns, non-significant; AUC, area under the curve; CI, confidence interval; TPR, true positive rate; FPR, False positive rate; blue circle: glucose, yellow circle: galactose, blue square: *N*-acetylglucosamine, yellow square: *N*-acetylgalactosamine, purple diamond: *N*-acetylneuraminic acid, red triangle: fucose. Related to Figure 3.

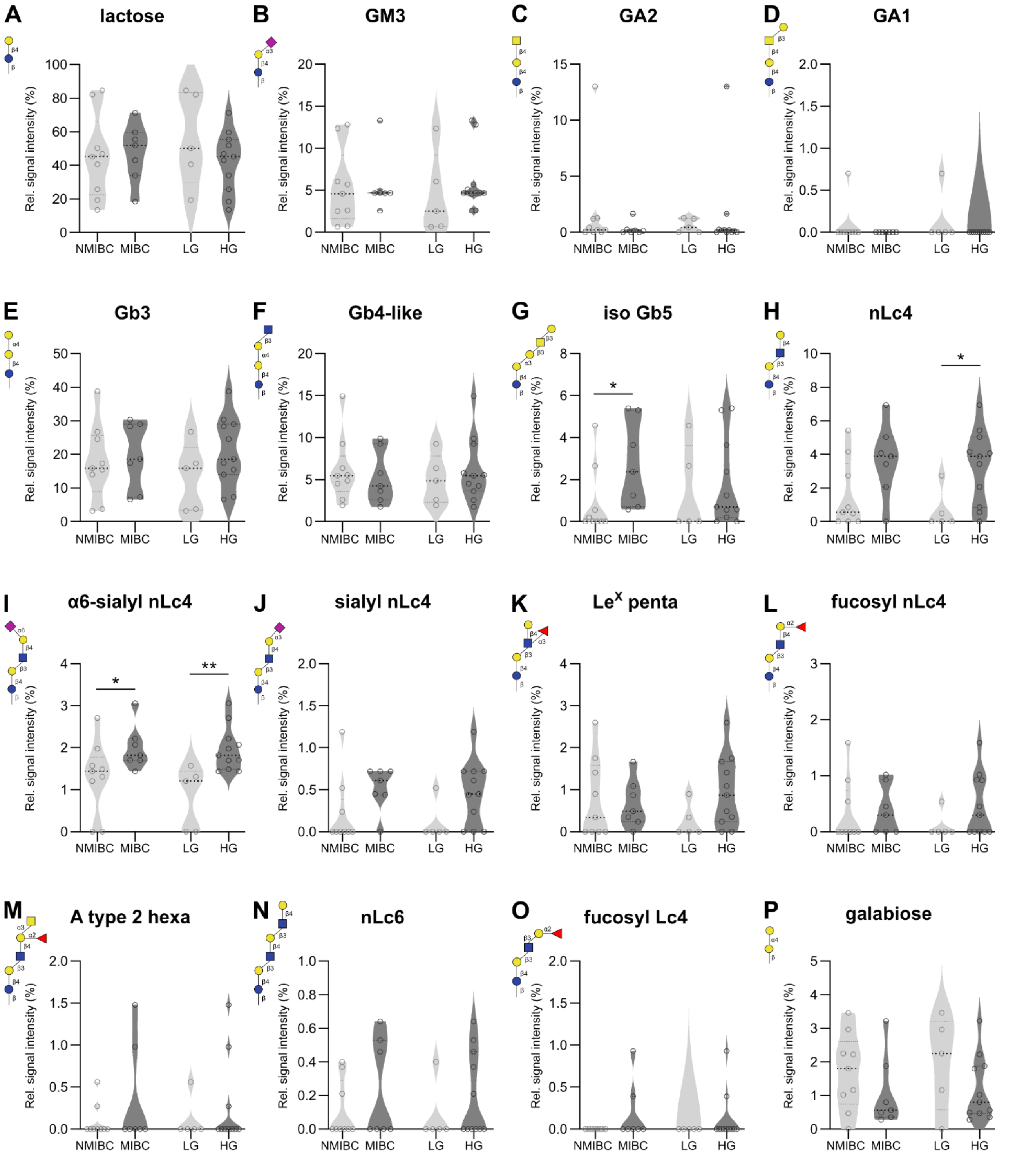

Figure S10. Glycosphingolipid profiling of urinary extracellular vesicles from bladder cancer patients in regard to tumor classification as NMIBC and MIBC and tumor grade. Related to Figure 4.

Glycosphingolipids detected in bladder cancer (BC, *n* = 16) urine samples through xCGE-LIF. Relative signal intensity levels of (A) lactose, (B) GM3, (C) GA2, (D) GA1, (E) Gb3, (F) Gb4-like, (G) iso Gb5, (H) nLc4, (I) α6-sialyl nLc4, (J) sialyl nLc4, (K) Le^{X} penta, (L) fucosyl nLc4, (M) A type 2 hexa, (N) nLc6, (O) fucosyl Lc4 and (P) galabiose across tumor pathological classification (left, NMIBC vs. MIBC) and disease grade (right, LG vs. HG). *p* values were calculated using two-tailed unpaired Mann-Whitney tests. Only statistically significant differences are represented. NMIBC, non-muscle invasive bladder cancer (*n* = 9); MIBC, muscle invasive bladder cancer (*n* = 7); LG, low grade (*n* = 5); HG, high grade (*n* = 11); *, *p* value <0.05; **, *p* value <0.005; blue circle: glucose, yellow circle: galactose, blue square: *N*-acetylglucosamine, yellow square: *N*-acetylgalactosamine, purple diamond: *N*-acetylneuraminic acid; red triangle: fucose.

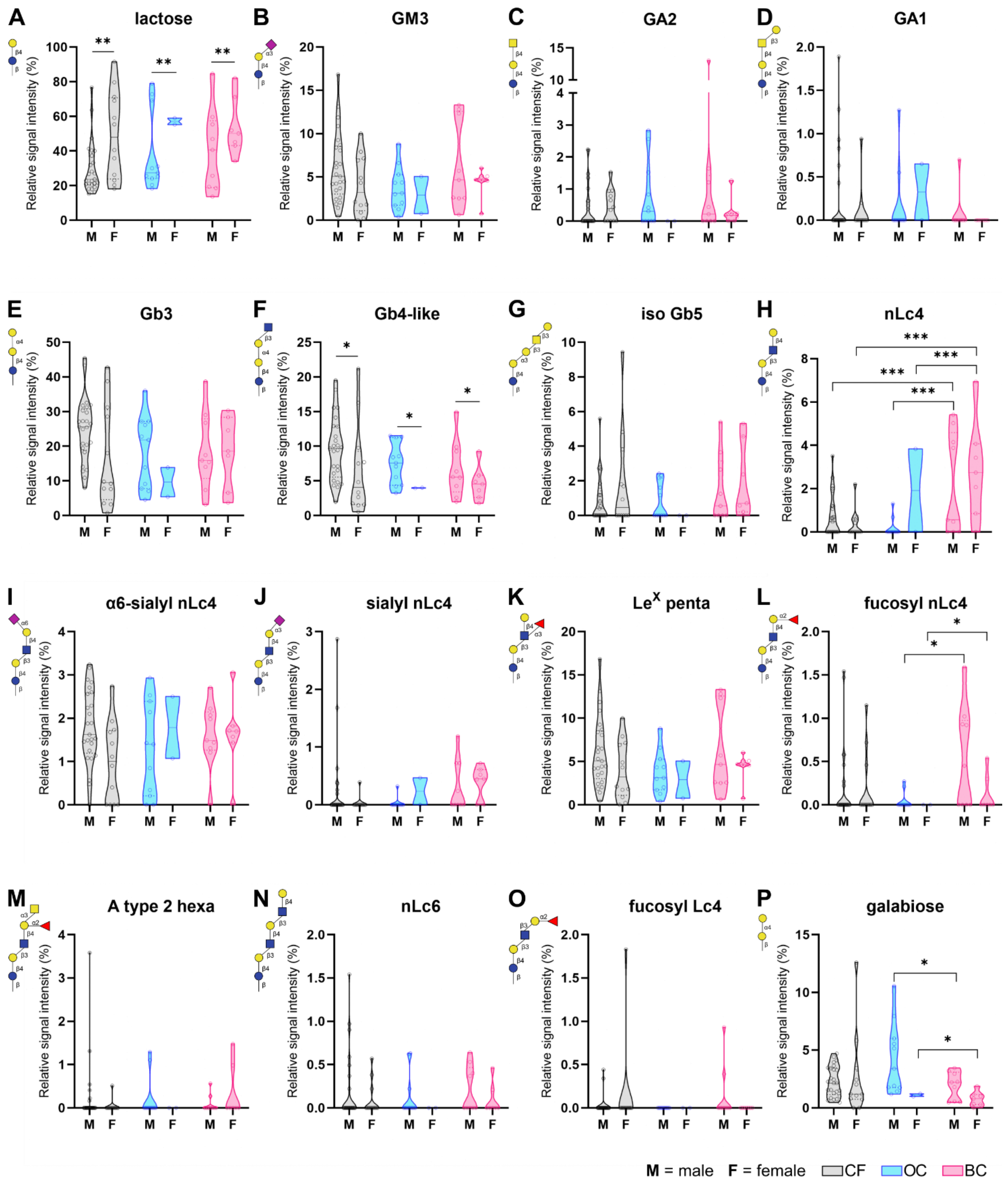

Figure S11. Glycosphingolipid profiling of urinary extracellular vesicles from bladder cancer patients across gender. **Related to Figure 4.**

Glycosphingolipids detected in urine of male and female individuals with bladder cancer (BC; males, n = 9; females, n = 7), cancer-free individuals (CF; males, n = 25; females, n = 12) and patients with other cancers (OC; males, n = 11; female n = 2) through xCGE-LIF. Violin plots reporting the relative signal intensity of (A) lactose, (B) GM3, (C) GA2, (D) GA1, (E) Gb3, (F) Gb4-like, (G) iso Gb5, (H) nLc4, (I) α6-sialyl nLc4, (J) sialyl nLc4, (K) Le^{X} penta, (L) fucosyl nLc4, (M) A type 2 hexa, (N) nLc6, (O) fucosyl Lc4 and (P) galabiose. p values were calculated using a 2way ANOVA test. Only statistically significant differences are represented. *, p value <0.05; **, p value <0.005; ***, p value <0.001; ns, non-significant; blue circle: glucose, yellow circle: galactose, blue square: *N*-acetylglucosamine, yellow square: *N*-acetylgalactosamine, purple diamond: *N*-acetylneuraminic acid, red triangle: fucose.

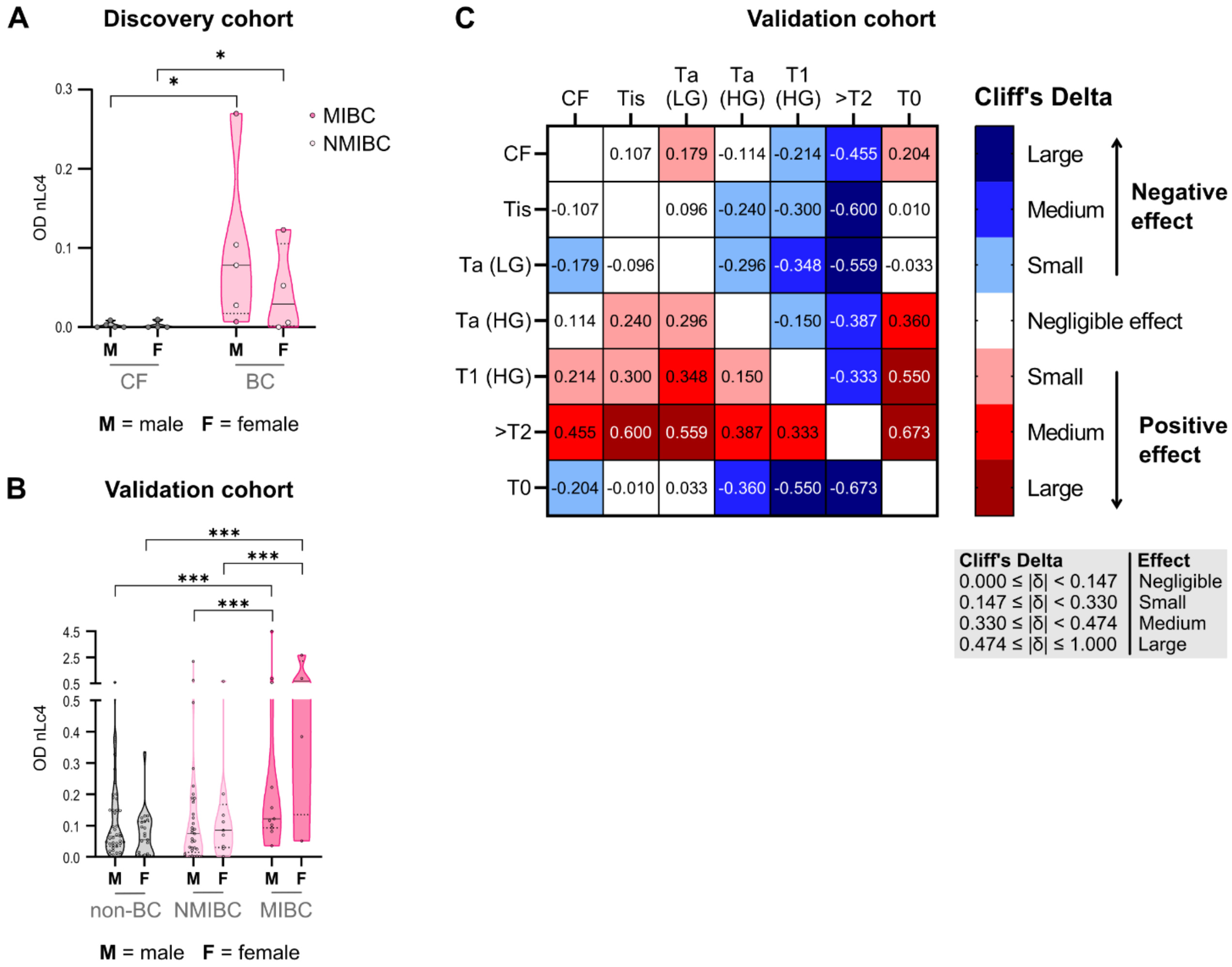

CF		Tis	Ta (LG)	Ta (HG)	T1 (HG)	>T2	T0
CF		0.107	0.179	-0.114	-0.214	-0.455	0.204
Tis	-0.107		0.096	-0.240	-0.300	-0.600	0.010
Ta (LG)	-0.179	-0.096		-0.296	-0.348	-0.559	-0.033
Ta (HG)	0.114	0.240	0.296		-0.150	-0.387	0.360
T1 (HG)	0.214	0.300	0.348	0.150		-0.333	0.550
>T2	0.455	0.600	0.559	0.387	0.333		0.673
T0	-0.204	-0.010	0.033	-0.360	-0.550	-0.673	

Cliff's Delta	Effect
0.000 ≤ |δ| < 0.147	Negligible
0.147 ≤ |δ| < 0.330	Small
0.330 ≤ |δ| < 0.474	Medium
0.474 ≤ |δ| ≤ 1.000	Large

Figure S12. nLc4 ELISA assay of urinary exosomes from bladder cancer patients across gender and tumor stage. Related to Figure 4.

(A-B) Violin plots reporting the optical density (OD) of nLc4 among patients of (A) the discovery cohort (CF; males, *n* = 5; females, *n* = 4. BC; males, *n* = 5; females, *n* = 4) and (B) the validation cohort (non-BC; males, *n* = 43; females, *n* = 23. NMIBC; males, *n* = 33; females, *n* = 9. MIBC; males, *n* = 11; females, *n* = 4). *p* values were calculated using a 2way ANOVA test. Only statistically significant differences are represented. *, *p* value <0.05; **, *p* value <0.005; ***, *p* value <0.001.

(C) Cliff's delta effect size matrix comparing nLc4 levels across different pathological stages of bladder cancer in the validation cohort. Each cell represents the Cliff's delta value for pairwise comparisons between groups, indicating the magnitude and direction of effect. Red indicates a positive effect (row group > column group), and blue a negative effect (column group > row group). Color intensity reflects effect size as shown in the legend. CF, cancer free; Tis, carcinoma *in situ*; HG, high grade; LG, low grade; T0, patients in remission.

A

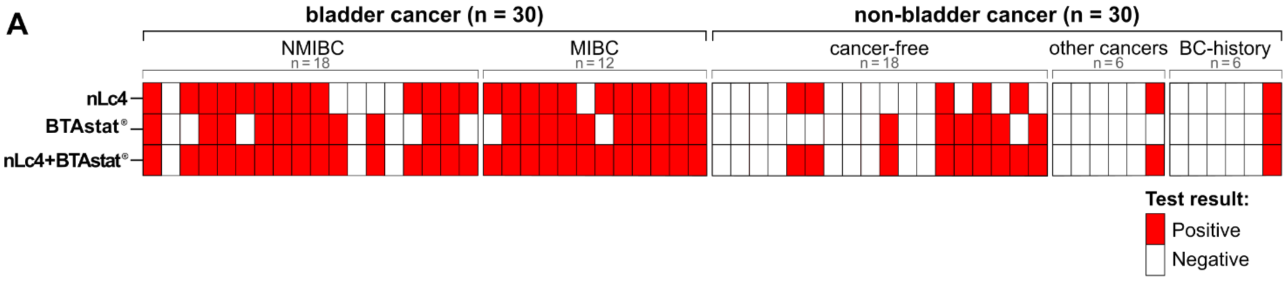

B

Test	Sensitivity	Specificity	PPV	NPV	Accuracy
nLc4	80.0%	**76.7%**	**77.4%**	79.3%	**78.3%**
BTAstat®	70.0%	**76.7%**	75.0%	71.9%	73.3%
nLc4 + BTAstat®	**90.0%**	63.3%	71.1%	**86.4%**	76.7%

Figure S13. Diagnostic performance of nLc4, and their combination in detecting bladder cancer from urine samples. Related Figure 4.

(A) Individual test results for nLc4, BTAstat® or nLc4 and BTAstat® tests combined across urine samples from bladder cancer (*n* = 30) and non-bladder cancer (*n* = 30) individuals. Each column represents a patient, and each row corresponds to one test. Red squares indicate a positive test result; white squares indicate a negative result. nLc4 levels were measured by a double-sandwich ELISA performed on urinary exosomes captured with the ExoTEST™ kit. A threshold of 0.08 optical density (OD) was applied; samples with OD ≥ 0.08 were considered positive. The combination (nLc4 + BTA *stat*) was considered positive when either test yielded a positive result. Samples are grouped into NMIBC (*n* = 18) and MIBC (*n* = 12) for bladder cancer, and into cancer-free (*n* = 18), other genitourinary/gynecologic cancers (*n* = 6), and patients with a history of bladder cancer (BC-history, *n* = 6) in the non-bladder cancer group.

(B) Sensitivity, specificity, positive predictive value (PPV), negative predictive value (NPV), and accuracy values of nLc4, BTAstat® and nLc4 and BTAstat® tests combined for bladder cancer diagnosis. Values are shown as percentages.

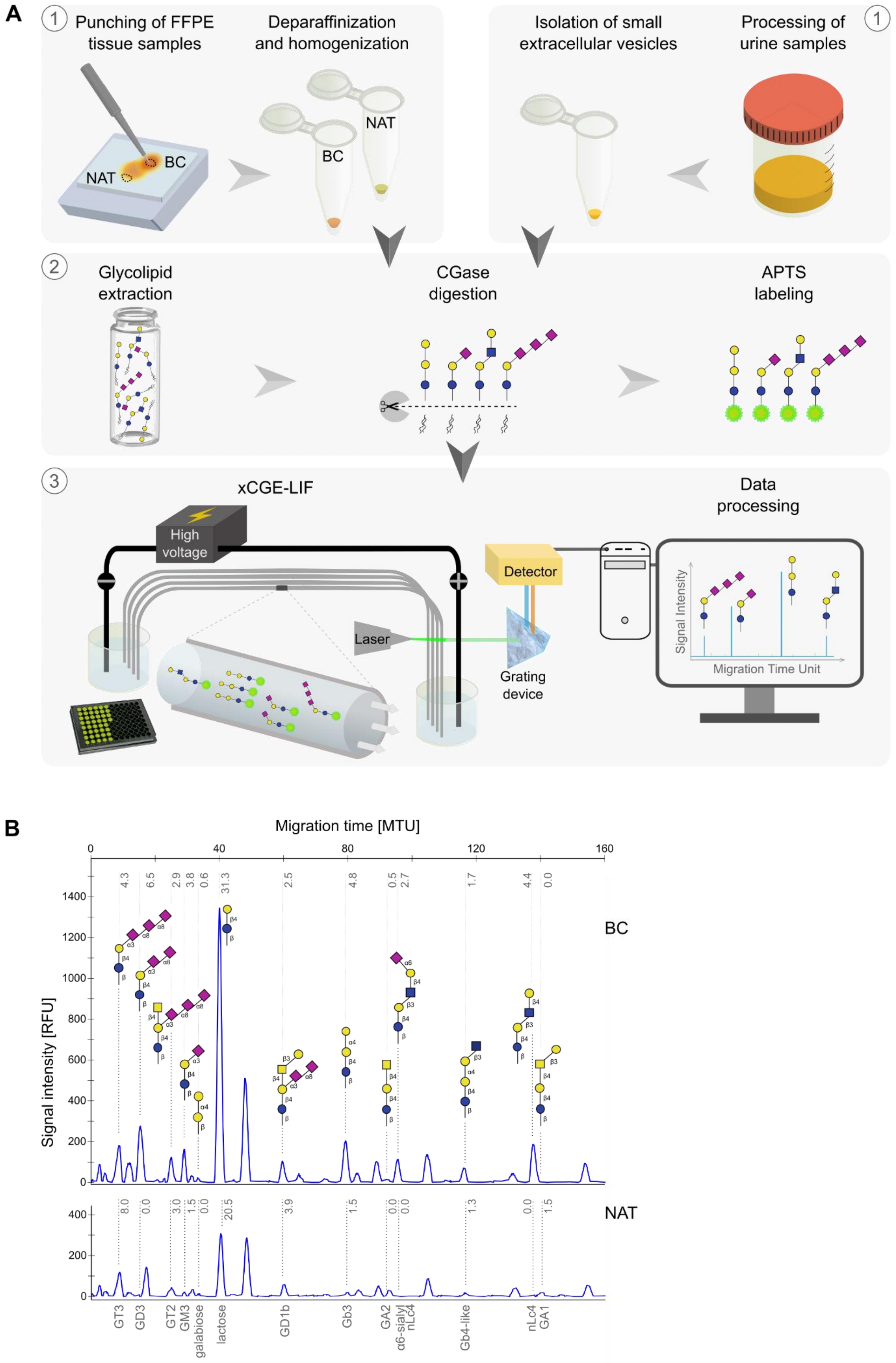

Figure S14. Schematic workflow for GSL-glycomics from punched FFPE tissues and urine samples and electropherograms profiles obtained. Related to STAR methods.

(A) Bladder tumor and normal adjacent tissue (NAT) regions of FFPE blocks were selected, punched and deparaffinized. 20 mL of urine samples were processed for small extracellular vesicles isolation through sequential centrifugation and ultracentrifugation steps. The tissue lysates and urinary small extracellular vesicle pellets obtained were used for GSL extraction, CGase digestion, APTS fluorescent labeling and purification for xCGE-LIF analysis. Labeled glycans were separated and monitored by xCGE-LIF, data were processed and glycans were annotated.
(B) Example of glycosphingolipid electropherogram profiles obtained from the bladder cancer and the normal adjacent tissue samples of patient number 24 from the tissue cohort (BC24, up; NAT24, down). Values in gray correspond to calculated relative signal intensities (in percentage). RFU, relative fluorescence units; MTU, migration time unit; Blue circle: glucose, yellow circle: galactose, blue square: *N*-acetylglucosamine, yellow square: *N*-acetylgalactosamine, purple diamond: *N*-acetylneuraminic acid.

Table S1. Clinical summary of the tissue, urine, discovery and validation cohorts. Related to Figures 1, 3 and 4.

IQR, interquartile range; NA, not applicable; NR, not reported; CIS, carcinoma in situ; MIBC, muscle invasive bladder cancer; NMIBC, non-muscle invasive bladder cancer.

	tissue cohort		urine cohort		discovery cohort		validation cohort	
**Groups**	**Patients** ***n*** **= 30**	**Controls** ***n*** **= 7**	**Patients** ***n*** **= 16**	**Controls** ***n*** **= 50**	**Patients** ***n*** **= 9**	**Controls** ***n*** **= 9**	**Patients** ***n*** **= 57**	**Controls** ***n*** **= 66**
**Gender**								
Male	27 (90%)	2 (29%)	9 (56%)	36 (72%)	5 (56%)	5 (56%)	44 (77%)	43 (%)
Female	3 (10%)	5 (71%)	7 (44%)	14 (28%)	4 (44%)	4 (44%)	13 (22%)	23 (%)
**Age in years**								
median (IQR)	69 (62.0-75.0)	63 (37.0-63.0)	73 (61.5-78.5)	60 (44.0-70.0)	76 (62.5-81.0)	57 (44.5-66.0)	70 (63.0-76.0)	69.5 (61.0-77.0)
**Clinical stage**								
CIS	4 (13%)	NA	1 (6%)	NA	0 (0%)	NA	5 (9%)	NA
Ta	1 (3%)	NA	7 (44%)	NA	6 (67%)	NA	33 (58%)	NA
T1	5 (17%)	NA	1 (6%)	NA	0 (0%)	NA	4 (7%)	NA
T2	9 (30%)	NA	2 (13%)	NA	1 (11%)	NA	13 (23%)	NA
T3	7 (23%)	NA	3 (19%)	NA	1 (11%)	NA	2 (3%)	NA
T4	4 (13%)	NA	2 (13%)	NA	1 (11%)	NA	0 (0%)	NA
**Tumor grade**								
Low grade	3 (10%)	NA	5 (31%)	NA	5 (56%)	NA	23 (40%)	NA
High grade	27 (90%)	NA	11 (69%)	NA	4 (44%)	NA	31 (55%)	NA
NR	0 (0%)	NA	0 (0%)	NA	0 (0%)	NA	3 (5%)	NA
**Classification**								
NMIBC	10 (33%)	NA	9 (56%)	NA	6 (67%)	NA	42 (74%)	NA
MIBC	20 (67%)	NA	7 (44%)	NA	3 (33%)	NA	15 (26%)	NA

*number of cases is shown for categorical variables with percentage in parentheses

Table S2. Individualized patient and tumor characteristics of the tissue cohort. Related to Figure 1.

NA, not applicable; BC, bladder cancer; NAT, normal adjacent tissue; CF, cancer-free; CIS, carcinoma *in situ*; yT, stage after neoadjuvant therapy; LG, low grade; HG, high grade; MIBC, muscle invasive bladder cancer; NMIBC, non-muscle invasive bladder cancer.

Sample ID	Patient number	Diagnosis	Age	Gender	Stage	Grade	Classification
NAT1	1	NAT	69	male	NA	NA	NA
BC1	1	BC	69	male	T3b	HG (G3)	MIBC
NAT2	2	NAT	59	female	NA	NA	NA
BC2	2	BC	59	female	T3b	HG (G3)	MIBC
NAT3	3	NAT	66	male	NA	NA	NA
BC3	3	BC	66	male	T2a	HG (G3)	MIBC
NAT4	4	NAT	69	male	NA	NA	NA
BC4	4	BC	69	male	T2b	HG (G2)	MIBC
NAT5	5	NAT	77	male	NA	NA	NA
BC5	5	BC	77	male	T1	HG (G2)	NMIBC
NAT6	6	NAT	73	male	NA	NA	NA
BC6	6	BC	73	male	T2b	HG (G3)	MIBC
NAT7	7	NAT	64	male	NA	NA	NA
BC7	7	BC	64	male	T4a	HG (G3)	MIBC
NAT8	8	NAT	79	male	NA	NA	NA
BC8	8	BC	79	male	T2a	HG (G2)	MIBC
NAT9	9	NAT	68	male	NA	NA	NA
BC9	9	BC	68	male	T3a	LG (G2)	MIBC
NAT10	10	NAT	78	male	NA	NA	NA
BC10	10	BC	78	male	T2b	HG (G3)	MIBC
NAT11	11	NAT	75	male	NA	NA	NA
BC11	11	BC	75	male	T4a	LG (G2)	MIBC
NAT12	12	NAT	62	female	NA	NA	NA
BC12	12	BC	62	female	T3b	HG (G3)	MIBC
NAT13	13	NAT	63	male	NA	NA	NA
BC13	13	BC	63	male	T1	HG (G3)	NMIBC
NAT14	14	NAT	48	male	NA	NA	NA
BC14	14	BC	48	male	T4b	HG (G3)	MIBC
NAT15	15	NAT	74	male	NA	NA	NA
BC15	15	BC	74	male	CIS	HG (CIS)	NMIBC
NAT16	16	NAT	74	male	NA	NA	NA
BC16	16	BC	74	male	T4	HG (G3)	MIBC
NAT17	17	NAT	76	male	NA	NA	NA
BC17	17	BC	76	male	T2a	HG (G2)	MIBC
NAT18	18	NAT	87	male	NA	NA	NA
BC18	18	BC	87	male	CIS	HG (CIS)	NMIBC
NAT19	19	NAT	70	male	NA	NA	NA
BC19	19	BC	70	male	T1	HG (G3)	NMIBC
NAT20	20	NAT	62	male	NA	NA	NA
BC20	20	BC	62	male	T1	HG (G3)	NMIBC
NAT21	21	NAT	67	male	NA	NA	NA
BC21	21	BC	67	male	T1	HG (G3)	NMIBC
NAT22	22	NAT	34	female	NA	NA	NA
BC22	22	BC	34	female	yT3a	HG (G3)	MIBC
NAT23	23	NAT	75	male	NA	NA	NA
BC23	23	BC	75	male	CIS	HG (CIS)	NMIBC

NAT24	24	NAT	87	male	NA	NA	NA
BC24	24	BC	87	male	T2b	HG (G3)	MIBC
NAT25	25	NAT	63	male	NA	NA	NA
BC25	25	BC	63	male	T2b	HG (G2)	MIBC
NAT26	26	NAT	74	male	NA	NA	NA
BC26	26	BC	74	male	T3b	HG (G3)	MIBC
NAT27	27	NAT	83	male	NA	NA	NA
BC27	27	BC	83	male	T3a	HG (G3)	MIBC
NAT28	28	NAT	50	male	NA	NA	NA
BC28	28	BC	50	male	yT2b	HG (G3)	MIBC
NAT29	29	NAT	57	male	NA	NA	NA
BC29	29	BC	57	male	CIS	HG (CIS)	NMIBC
NAT30	30	NAT	47	male	NA	NA	NA
BC30	30	BC	47	male	Ta	LG (G2)	NMIBC
CF1	31	CF	25	female	NA	NA	NA
CF2	32	CF	63	female	NA	NA	NA
CF3	33	CF	75	female	NA	NA	NA
CF4	34	CF	63	male	NA	NA	NA
CF5	35	CF	58	male	NA	NA	NA
CF6	36	CF	63	female	NA	NA	NA
CF7	37	CF	37	female	NA	NA	NA

Table S3. Individualized patient and tumor characteristics of the urine cohort. Subjects of the discovery cohort are included. Related to Figures 3 and 4.

NA, not applicable; BC, bladder cancer; CF, cancer-free; OC, other cancers; CIS, carcinoma *in situ*; LG, low grade; HG, high grade; MIBC, muscle invasive bladder cancer; NMIBC, non-muscle invasive bladder cancer.

Sample ID	Patient number	Diagnosis	Group	Age	Gender	Stage	Grade	Classification	Sample type collected	ELISA (discovery cohort)	BTA Stat result
BC1	1	Bladder cancer	BC	73	male	T2b	HG (G3)	MIBC	urine, tissue	No	-
BC2	2	Bladder cancer	BC	73	male	T1	HG (G3)	NMIBC	urine, tissue	No	-
BC3	3	Bladder cancer	BC	79	male	Ta	LG (G2)	NMIBC	urine, tissue	Yes	Negative
BC4	4	Bladder cancer	BC	68	male	Ta	LG (G2)	NMIBC	urine, tissue	Yes	Negative
BC5	5	Bladder cancer	BC	66	female	CIS	HG (CIS)	NMIBC	urine, tissue	No	-
BC6	6	Bladder cancer	BC	51	male	Ta	HG (G3)	NMIBC	urine, tissue	Yes	Positive
BC7	7	Bladder cancer	BC	83	female	Ta	LG (G1)	NMIBC	urine, tissue	Yes	Negative
BC8	8	Bladder cancer	BC	76	female	T2	HG (G2)	MIBC	urine, tissue	Yes	Positive
BC9	9	Bladder cancer	BC	57	male	Ta	LG (G2)	NMIBC	urine, tissue	Yes	Negative
BC10	10	Bladder cancer	BC	78	female	T3b	HG (G3)	MIBC	urine	Yes	Positive
BC11	11	Bladder cancer	BC	69	male	T4a	HG (G3)	MIBC	urine	Yes	Positive
BC12	12	Bladder cancer	BC	82	female	Ta	LG (G2)	NMIBC	urine	Yes	Positive
BC13	13	Bladder cancer	BC	79	female	T3a	HG (G3)	MIBC	urine, tissue	No	-
BC14	14	Bladder cancer	BC	78	male	T3a	HG (G3)	MIBC	urine, tissue	No	-
BC15	15	Bladder cancer	BC	56	female	T4a	HG (G3)	MIBC	urine, tissue	No	-
BC16	16	Bladder cancer	BC	55	male	Ta	HG (G2)	NMIBC	urine, tissue	No	-
OC1	17	Prostate cancer	non-BC	64	male	NA	NA	NA	urine	No	-
OC2	18	Endometrial cancer	non-BC	70	female	NA	NA	NA	urine	No	-
OC3	19	Gynecologic cancer	non-BC	31	female	NA	NA	NA	urine	No	-
OC4	20	Melanoma of the bladder	non-BC	78	male	NA	NA	NA	urine	No	-
OC5	21	Prostate cancer	non-BC	60	male	NA	NA	NA	urine	No	-
OC6	22	Prostate cancer	non-BC	67	male	NA	NA	NA	urine	No	-
OC7	23	Urothelial carcinoma of the upper urinary tract	non-BC	78	male	NA	NA	NA	urine	No	-
OC8	24	Renal cell carcinoma	non-BC	58	male	NA	NA	NA	urine	No	-
OC9	25	Prostate cancer	non-BC	68	male	NA	NA	NA	urine	No	-
OC10	26	Renal cell carcinoma	non-BC	65	male	NA	NA	NA	urine	No	-
OC11	27	Renal cell carcinoma	non-BC	58	male	NA	NA	NA	urine	No	-
OC12	28	Prostate cancer	non-BC	49	male	NA	NA	NA	urine	No	-
OC13	29	Prostate cancer	non-BC	72	male	NA	NA	NA	urine	No	-
CF1	30	Cancer-free	non-BC	72	male	NA	NA	NA	urine	No	-
CF2	31	Cancer-free	non-BC	67	male	NA	NA	NA	urine	No	-

CF3	32	Cancer-free	non-BC	77	male	NA	NA	NA	urine	No	-
CF4	33	Cancer-free	non-BC	21	male	NA	NA	NA	urine	No	-
CF5	34	Cancer-free	non-BC	58	male	NA	NA	NA	urine	No	-
CF6	35	Cancer-free	non-BC	53	male	NA	NA	NA	urine	Yes	Negative
CF7	36	Cancer-free	non-BC	50	male	NA	NA	NA	urine	Yes	Negative
CF8	37	Cancer-free	non-BC	72	male	NA	NA	NA	urine	No	-
CF9	38	Cancer-free	non-BC	38	male	NA	NA	NA	urine	No	-
CF10	39	Cancer-free	non-BC	57	male	NA	NA	NA	urine	Yes	Negative
CF11	40	Cancer-free	non-BC	36	female	NA	NA	NA	urine	No	-
CF12	41	Cancer-free	non-BC	62	male	NA	NA	NA	urine	Yes	Negative
CF13	42	Cancer-free	non-BC	69	female	NA	NA	NA	urine	Yes	Negative
CF14	43	Cancer-free	non-BC	36	female	NA	NA	NA	urine	Yes	Negative
CF15	44	Cancer-free	non-BC	39	female	NA	NA	NA	urine	Yes	Negative
CF16	45	Cancer-free	non-BC	86	female	NA	NA	NA	urine	No	-
CF17	46	Cancer-free	non-BC	44	male	NA	NA	NA	urine	No	-
CF18	47	Cancer-free	non-BC	68	male	NA	NA	NA	urine	No	-
CF19	48	Cancer-free	non-BC	60	male	NA	NA	NA	urine	No	-
CF20	49	Cancer-free	non-BC	75	female	NA	NA	NA	urine	No	-
CF21	50	Cancer-free	non-BC	66	male	NA	NA	NA	urine	No	-
CF22	51	Cancer-free	non-BC	62	male	NA	NA	NA	urine	No	-
CF23	52	Cancer-free	non-BC	60	female	NA	NA	NA	urine	Yes	Negative
CF24	53	Cancer-free	non-BC	43	female	NA	NA	NA	urine	No	-
CF25	54	Cancer-free	non-BC	69	male	NA	NA	NA	urine	No	-
CF26	55	Cancer-free	non-BC	54	male	NA	NA	NA	urine	No	-
CF27	56	Cancer-free	non-BC	55	female	NA	NA	NA	urine	No	-
CF28	57	Cancer-free	non-BC	77	male	NA	NA	NA	urine	Yes	Negative
CF29	58	Cancer-free	non-BC	56	male	NA	NA	NA	urine	No	-
CF30	59	Cancer-free	non-BC	77	male	NA	NA	NA	urine	No	-
CF31	60	Cancer-free	non-BC	25	female	NA	NA	NA	urine	No	-
CF32	61	Cancer-free	non-BC	27	female	NA	NA	NA	urine	No	-
CF33	62	Cancer-free	non-BC	39	male	NA	NA	NA	urine	No	-
CF34	63	Cancer-free	non-BC	27	female	NA	NA	NA	urine	No	-
CF35	64	Cancer-free	non-BC	29	male	NA	NA	NA	urine	No	-
CF36	65	Cancer-free	non-BC	79	male	NA	NA	NA	urine	No	-
CF37	66	Cancer-free	non-BC	75	male	NA	NA	NA	urine	No	-

Table S4. Individualized patient and tumor characteristics of the validation cohort. Related to Figure 4.

BC, bladder cancer; BC-H, bladder cancer history; LG, low grade; HG, high grade; KRH, Klinikum Region Hannover; MIBC, muscle invasive bladder cancer; NMIBC, non-muscle invasive bladder cancer; NA, not applicable; NR, not reported; pT, pathological staging based on histological examination; T, clinical staging where histological confirmation was not available UKJ, Universitätsklinikum Jena; UKT, Universitätsklinikum Tübingen; ypT, stage after neoadjuvant therapy.

Sample ID	Patient number	Diagnosis	Group	Age	Gender	Stage	Grade	Classification	Hospital	BTA Stat result
BC1	1	Bladder cancer	BC	83	male	Ta	LG	NMIBC	KRH Siloah	-
BC2	2	Bladder cancer	BC	55	male	Ta	LG	NMIBC	KRH Siloah	-
BC3	3	Bladder cancer	BC	64	male	Ta	LG	NMIBC	KRH Siloah	-
BC4	4	Bladder cancer	BC	84	male	Ta	LG	NMIBC	KRH Siloah	-
BC5	5	Bladder cancer	BC	71	male	Ta	LG	NMIBC	KRH Siloah	-
BC6	6	Bladder cancer	BC	62	female	Ta	LG	NMIBC	KRH Siloah	-
BC7	7	Bladder cancer	BC	66	male	Ta	HG	NMIBC	KRH Siloah	-
BC8	8	Bladder cancer	BC	75	female	pTa	HG	NMIBC	KRH Siloah	-
BC9	9	Bladder cancer	BC	76	male	pTa	LG	NMIBC	KRH Siloah	-
BC10	10	Bladder cancer	BC	79	male	pT2a	HG	MIBC	KRH Siloah	-
BC11	11	Bladder cancer	BC	61	female	Ta	LG	NMIBC	KRH Siloah	-
BC12	12	Bladder cancer	BC	42	male	pTa	LG	NMIBC	KRH Siloah	-
BC13	13	Bladder cancer	BC	70	male	pT2a	HG	MIBC	KRH Siloah	-
BC14	14	Bladder cancer	BC	74	male	pTa	HG	NMIBC	KRH Siloah	-
BC15	15	Bladder cancer	BC	77	male	pTa	LG	NMIBC	KRH Siloah	-
BC16	16	Bladder cancer	BC	60	female	T2a	HG	MIBC	KRH Siloah	-
BC17	17	Bladder cancer	BC	68	female	T2a	HG (G3)	MIBC	UKJ	-
BC18	18	Bladder cancer	BC	76	male	T1	HG	NMIBC	UKJ	Positive
BC19	19	Bladder cancer	BC	85	male	Ta	LG	NMIBC	UKJ	-
BC20	20	Bladder cancer	BC	71	male	Ta	HG	NMIBC	UKJ	Positive
BC21	21	Bladder cancer	BC	79	male	T1	HG (G2)	NMIBC	UKJ	Positive
BC22	22	Bladder cancer	BC	38	male	yT2a	NR	MIBC	UKJ	-
BC23	23	Bladder cancer	BC	68	female	Ta	LG	NMIBC	UKJ	Positive
BC24	24	Bladder cancer	BC	70	male	T2a	HG (G2)	MIBC	UKJ	Positive
BC25	25	Bladder cancer	BC	81	male	T1	HG (G3)	NMIBC	UKJ	-
BC26	26	Bladder cancer	BC	68	female	Ta	HG	NMIBC	UKJ	-
BC27	27	Bladder cancer	BC	67	male	T2a	HG (G3)	MIBC	UKJ	Positive
BC28	28	Bladder cancer	BC	73	male	Ta	LG (G2)	NMIBC	UKJ	Positive
BC29	29	Bladder cancer	BC	65	male	T1	HG	NMIBC	UKJ	-
BC30	30	Bladder cancer	BC	74	male	Ta	LG (G1)	NMIBC	UKJ	-
BC31	31	Bladder cancer	BC	76	male	T2a	NR	MIBC	UKJ	-
BC32	32	Bladder cancer	BC	75	male	T2	HG (G3)	MIBC	UKJ	Positive
BC33	33	Bladder cancer	BC	32	female	Ta	LG	NMIBC	UKJ	Negative
BC34	34	Bladder cancer	BC	68	male	CIS	HG (G3)	NMIBC	UKJ	Positive
BC35	35	Bladder cancer	BC	36	female	Ta	LG (G1)	NMIBC	UKJ	Negative
BC36	36	Bladder cancer	BC	76	male	CIS	HG (G3)	NMIBC	UKJ	-
BC37	37	Bladder cancer	BC	65	male	Ta	LG (G2)	NMIBC	UKJ	-
BC38	38	Bladder cancer	BC	84	male	Ta	LG (G2)	NMIBC	UKJ	-

BC39	39	Bladder cancer	BC	71	male	Ta	LG (G1)	NMIBC	UKJ	-
BC40	40	Bladder cancer	BC	81	male	Ta	LG (G1)	NMIBC	UKJ	-
BC41	41	Bladder cancer	BC	57	male	yTa	LG (G2)	NMIBC	UKT	Negative
BC42	42	Bladder cancer	BC	61	female	yT2a	HG (G3)	MIBC	UKT	Negative
BC43	43	Bladder cancer	BC	62	male	T2a	HG (G3)	MIBC	UKT	Positive
BC44	44	Bladder cancer	BC	68	female	yT3b	HG (G3)	MIBC	UKT	Positive
BC45	45	Bladder cancer	BC	83	female	CIS	HG (G3)	NMIBC	UKT	Positive
BC46	46	Bladder cancer	BC	65	male	Ta	HG (G2)	NMIBC	UKT	-
BC47	47	Bladder cancer	BC	69	male	T2a	NR	MIBC	UKT	Positive
BC48	48	Bladder cancer	BC	73	male	CIS	HG (G3)	NMIBC	UKT	Positive
BC49	49	Bladder cancer	BC	54	male	T3a	HG (G3)	MIBC	UKT	Positive
BC50	50	Bladder cancer	BC	83	male	Ta	HG (G3)	NMIBC	UKT	Negative
BC51	51	Bladder cancer	BC	75	male	T2a	HG (G3)	MIBC	UKT	Positive
BC52	52	Bladder cancer	BC	85	female	Ta	HG (G2)	NMIBC	UKT	-
BC53	53	Bladder cancer	BC	38	male	Ta	LG (G1)	NMIBC	UKT	-
BC54	54	Bladder cancer	BC	69	male	CIS	HG (G3)	NMIBC	UKT	-
BC55	55	Bladder cancer	BC	75	male	Ta	HG (G2)	NMIBC	UKT	-
BC56	56	Bladder cancer	BC	58	male	Ta	LG (G2)	NMIBC	UKT	-
BC57	57	Bladder cancer	BC	78	male	Ta	HG (G3)	NMIBC	UKT	-
CF1	58	Cancer-free	non-BC	61	female	NA	NA	NA	KRH Siloah	-
CF2	59	Cancer-free	non-BC	58	male	NA	NA	NA	KRH Siloah	-
CF3	60	Cancer-free	non-BC	53	male	NA	NA	NA	KRH Siloah	-
CF4	61	Cancer-free	non-BC	84	female	NA	NA	NA	KRH Siloah	-
CF5	62	Cancer-free	non-BC	79	male	NA	NA	NA	KRH Siloah	-
CF6	63	Cancer-free	non-BC	60	male	NA	NA	NA	KRH Siloah	-
CF7	64	Cancer-free	non-BC	62	male	NA	NA	NA	KRH Siloah	-
CF8	65	Cancer-free	non-BC	62	female	NA	NA	NA	UKJ	-
CF9	66	Cancer-free	non-BC	79	female	NA	NA	NA	UKJ	Positive
CF10	67	Cancer-free	non-BC	69	female	NA	NA	NA	UKJ	Negative
CF11	68	Cancer-free	non-BC	71	female	NA	NA	NA	UKJ	-
CF12	69	Cancer-free	non-BC	71	female	NA	NA	NA	UKJ	-
CF13	70	Cancer-free	non-BC	57	male	NA	NA	NA	UKJ	Negative
CF14	71	Cancer-free	non-BC	87	female	NA	NA	NA	UKJ	-
CF15	72	Cancer-free	non-BC	70	female	NA	NA	NA	UKJ	Positive
CF16	73	Cancer-free	non-BC	68	male	NA	NA	NA	UKJ	-
CF17	74	Cancer-free	non-BC	44	female	NA	NA	NA	UKJ	-
CF18	75	Cancer-free	non-BC	53	female	NA	NA	NA	UKJ	-
CF19	76	Cancer-free	non-BC	71	male	NA	NA	NA	UKJ	Positive
CF20	77	Cancer-free	non-BC	82	male	NA	NA	NA	UKJ	-
CF21	78	Cancer-free	non-BC	79	male	NA	NA	NA	UKJ	Positive
CF22	79	Cancer-free	non-BC	71	male	NA	NA	NA	UKJ	Positive
CF23	80	Cancer-free	non-BC	54	female	NA	NA	NA	UKJ	Negative
CF24	81	Cancer-free	non-BC	81	male	NA	NA	NA	UKJ	-
CF25	82	Cancer-free	non-BC	55	female	NA	NA	NA	UKJ	Positive
CF26	83	Cancer-free	non-BC	76	male	NA	NA	NA	UKT	-

CF27	84	Cancer-free	non-BC	70	male	NA	NA	NA	UKT	-
CF28	85	Cancer-free	non-BC	69	female	NA	NA	NA	UKT	-
BC-H1	86	Bladder cancer history	non-BC	87	female	NA	NA	NA	UKJ	-
BC-H2	87	Bladder cancer history	non-BC	72	female	NA	NA	NA	UKJ	-
BC-H3	88	Bladder cancer history	non-BC	75	female	NA	NA	NA	UKJ	Negative
BC-H4	89	Bladder cancer history	non-BC	85	male	NA	NA	NA	UKJ	-
BC-H5	90	Bladder cancer history	non-BC	84	male	NA	NA	NA	UKJ	Negative
BC-H6	91	Bladder cancer history	non-BC	92	female	NA	NA	NA	UKJ	Negative
BC-H7	92	Bladder cancer history	non-BC	67	male	NA	NA	NA	UKJ	-
BC-H8	93	Bladder cancer history	non-BC	81	male	NA	NA	NA	UKJ	-
BC-H9	94	Bladder cancer history	non-BC	78	male	NA	NA	NA	UKJ	-
BC-H10	95	Bladder cancer history	non-BC	73	female	NA	NA	NA	UKJ	-
BC-H11	96	Bladder cancer history	non-BC	58	male	NA	NA	NA	UKJ	-
BC-H12	97	Bladder cancer history	non-BC	63	male	NA	NA	NA	UKJ	-
BC-H13	98	Bladder cancer history	non-BC	47	male	NA	NA	NA	UKJ	Negative
BC-H14	99	Bladder cancer history	non-BC	70	male	NA	NA	NA	UKJ	-
BC-H15	100	Bladder cancer history	non-BC	72	male	NA	NA	NA	UKJ	-
BC-H16	101	Bladder cancer history	non-BC	68	male	NA	NA	NA	UKJ	-
BC-H17	102	Bladder cancer history	non-BC	62	male	NA	NA	NA	UKJ	-
BC-H18	103	Bladder cancer history	non-BC	64	male	NA	NA	NA	UKJ	-
BC-H19	104	Bladder cancer history	non-BC	77	male	NA	NA	NA	UKJ	Negative
BC-H20	105	Bladder cancer history	non-BC	67	male	NA	NA	NA	UKJ	Positive
OC1	106	Other cancer	non-BC	51	male	NA	NA	NA	KRH Siloah	-
OC2	107	Prostate cancer	non-BC	83	male	NA	NA	NA	UKJ	Negative
OC3	108	Ovarian cancer	non-BC	58	female	NA	NA	NA	UKJ	Negative
OC4	109	Renal cell carcinoma	non-BC	51	female	NA	NA	NA	UKT	Negative
OC5	110	Renal cell carcinoma	non-BC	68	female	NA	NA	NA	UKT	-
OC6	111	Renal cell carcinoma	non-BC	58	male	NA	NA	NA	UKT	Negative
OC7	112	Renal cell carcinoma	non-BC	60	male	NA	NA	NA	UKT	-
OC8	113	Renal cell carcinoma	non-BC	78	male	NA	NA	NA	UKT	-
OC9	114	Renal cell carcinoma	non-BC	82	male	NA	NA	NA	UKT	-
OC10	115	Renal cell carcinoma	non-BC	61	male	NA	NA	NA	UKT	Negative
OC11	116	Renal cell carcinoma	non-BC	61	male	NA	NA	NA	UKT	-
OC12	117	Renal cell carcinoma	non-BC	77	male	NA	NA	NA	UKT	-
OC13	118	Renal cell carcinoma	non-BC	70	male	NA	NA	NA	UKT	-
OC14	119	Renal cell carcinoma	non-BC	68	female	NA	NA	NA	UKT	-
OC15	120	Renal cell carcinoma	non-BC	73	male	NA	NA	NA	UKT	Negative
OC16	121	Renal cell carcinoma	non-BC	67	male	NA	NA	NA	UKT	-
OC17	122	Renal cell carcinoma	non-BC	70	male	NA	NA	NA	UKT	-
OC18	123	Renal cell carcinoma	non-BC	46	male	NA	NA	NA	UKT	-

Table S5. Information and structure of all the GSL glycans detected through xCGE-LIF in this study. Related to Figures 1, 3 and 4.

Blue circle: glucose, yellow circle: galactose, blue square: *N*-acetylglucosamine, yellow square: *N*-acetylgalactosamine, purple diamond: *N*-acetylneuraminic acid, red triangle: fucose.

Common name	Name	Composition	Category	Structure
lactose	Lactosylceramide	Galβ1-4Glcβ-Cer	–	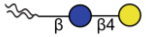
galabiose	Galabiosylceramide	Galα1-4Galβ-Cer	gala-series	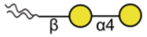
GM3	Monosialodihexosylganglioside	NeuAcα2-3Galβ1-4Glcβ-Cer	ganglio-series	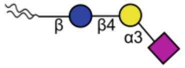
GD3	Disialodihexosylganglioside	NeuAcα2-8NeuAcα2-3Galβ1-4Glcβ-Cer	ganglio-series	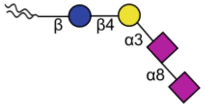
GD1b	Disialotetrahexosylganglioside	NeuAcα2-8NeuAcα2-3(Galβ1-3GalNAcβ1-4)Galβ1--4Glcβ-Cer	ganglio-series	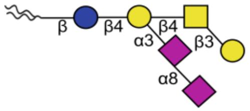
GT3	Trisialodihexosylganglioside	NeuAcα2-8NeuAcα2-8NeuAcα2-3Galβ1-4Glcβ-Cer	ganglio-series	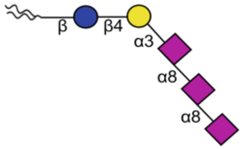
GT2	Trisialotrihexosylganglioside	NeuAcα2-8NeuAcα2-8NeuAcα2-3(GalNAcβ1-4)Galβ1--4Glcβ-Cer	ganglio-series	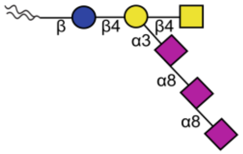
GA2	Asialo-monosialotrihexosylganglioside (asialo-GM2)	GalNAcβ1-4Galβ1-4Glcβ-Cer	asialo-ganglio-series	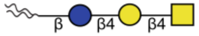
GA1	Asialo-monosialotetrahexosylganglioside (asialo-GM1)	Galβ1-3GalNAcβ1-4Galβ1-4Glcβ-Cer	asialo-ganglio-series	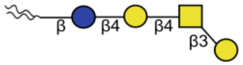
Gb3	Globotriaosylceramide	Galα1-4Galβ1-4Glcβ-Cer	globo-series	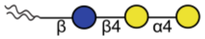
Gb4-like	Globolactotetraosylceramide-like	GlcNAcβ1-3Galα1-4Galβ1-4Glcβ-Cer	globo-series	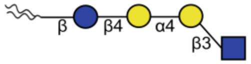
iso Gb5	Isoglobopentaosylceramide	Galβ1-3GalNAcβ1-3Galα1-3Galβ1-4Glcβ-Cer	isoglobo-series	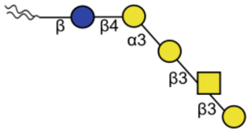

fucosyl Lc4	Lacto-*N*-fucopentaosylceramide	Fucα1-2Galβ1-3GlcNAcβ1-3Galβ1-4Glcβ-Cer	lacto-series	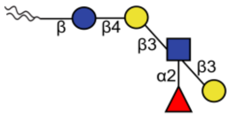
nLc4	Neolactotetraosylceramide	Galβ1-4GalNAcβ1-3Galβ1-4Glcβ-Cer	neolacto-series	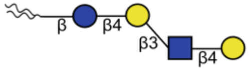
sialyl nLc4	Sialosylneolactotetraosylceramide	NeuAcα2-3Galβ1-4GalNAcβ1-3Galβ1-4Glcβ-Cer	neolacto-series	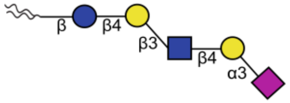
α6-sialyl nLc4	Sialosyl-6-neolactotetraosylceramide	NeuAcα2-6Galβ1-4GalNAcβ1-3Galβ1-4Glcβ-Cer	neolacto-series	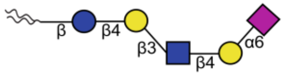
Le^{X} penta	Lewis X pentaosylceramide	Fucα1-3(Galβ1-4)GlcNAcβ1-3Galβ1-4Glcβ-Cer	neolacto-series	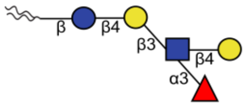
fucosyl nLc4	Fucosylneolactotetraosylceramide	Fucα1-2Galβ1-4GlcNAcβ1-3Galβ1-4Glcβ-Cer	neolacto-series	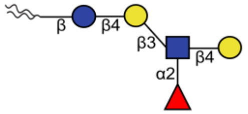
A type 2 hexa	Blood group A antigen hexaosylceramide type 2	GalNAcα1-3(Fucα1-2)Galβ1-4GlcNAcβ1-3Galβ1--4Glcβ-Cer	neolacto-series	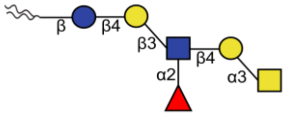
nLc6	Neolactohexaosylceramide	Galβ1-4GlcNAcβ1-3Galβ1-4GlcNAcβ1-3Galβ1--4Glcβ-Cer	neolacto-series	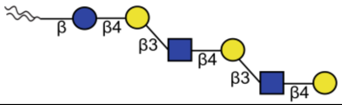
